# The SaeRS two-component system likely mediates the major dopamine-dependent regulation of virulence in *Staphylococcus aureus* ATCC 25,923

**DOI:** 10.1186/s12866-026-05427-4

**Published:** 2026-08-18

**Authors:** Ben Xu, Ziyue Wang, Jinmei Chai, Yunlin Wei

**Affiliations:** Faculty of Life Science and Technology, Kunming University of Science and Technology, Kunming, 650500 China

**Keywords:** *Staphylococcus aureus*, Dopamine, catecholamine, SaeRS, Microbial endocrinology, Virulence, Two-component system, Quorum sensing

## Abstract

*Staphylococcus aureus*(*S. aureus*)is an important foodborne pathogen. Host-derived catecholamines released under stress are known to influence bacterial pathogenic processes, including growth, virulence, biofilm formation. However, the role of dopamine in this context remains less well understood, particularly in *S. aureus*. In this study, we investigated the effects of dopamine on *S. aureus* ATCC 25,923 using a combination of phenotypic assays and multi-omics analyses. Dopamine was taken up by the bacteria and was associated with enhanced pathogenic phenotypes, including increased proliferation, toxin production, biofilm formation, motility, adhesion, and multidrug resistance. In a mouse infection experiment, dopamine treatment further increased bacterial colonization and organ invasion. Mechanistically, multi-omics analyses indicated that dopamine activates the SaeRS two-component system and is associated with the upregulation of virulence-related factors, adhesion-associated proteins, and membrane lipid synthesis pathways, along with increased activity in key metabolic processes such as the tricarboxylic acid (TCA) cycle and oxidative phosphorylation. Importantly, deletion of *saeS* markedly attenuated these dopamine-associated effects. Taken together, these findings suggest that dopamine likely influences *S. aureus* pathogenicity primarily through the SaeRS signaling pathway and provide insight into how host stress signals may contribute to bacterial infection.

**Supplementary Information:**

The online version contains supplementary material available at https://doi.org/10.1186/s12866-026-05427-4.

## Introduction

*S. aureus* is a common foodborne pathogen and a Gram-positive bacterium. It is frequently present on human skin and mucous membranes. It can cause food poisoning, sepsis, and a range of inflammatory conditions [1, 2]. This organism mainly induces disease through the production of toxins and invasive enzymes. Its virulence factors include hemolysins, leukocidins, and enterotoxins. They also encompass coagulase and phenol-soluble regulatory proteins [3, 4].

Since the early 20th century, purified catecholamines—including epinephrine(EPI), norepinephrine༈NE༉, and dopamine༈DA༉—have been widely applied in clinical emergency care and in the management of shock [5]. Their primary effects involve the regulation of cardiovascular function and also elevate blood pressure and improve tissue perfusion [6]. Clinicians have noted that increased catecholamine levels in trauma patients are closely associated with severe bacterial infections. These elevations can also precipitate serious infectious conditions, including sepsis [7].Subsequent studies have further demonstrated that catecholamine neurotransmitters can significantly promote the growth rate of pathogenic bacteria such as *Escherichia coli*༈*E. coli*༉and *Pseudomonas aeruginosa*, enhance swimming and swarming motility, increase biofilm formation, and globally upregulate the expression of virulence‑related regulatory genes [8]; they also markedly elevate the tolerance of these strains to multiple clinically used antibiotics [9].

Two potential mechanisms may account for this phenomenon. One hypothesis is that catecholamines facilitate bacterial iron acquisition through the ability of their catechol moiety to chelate ferric iron [10]. In this way, they facilitate pathogen iron uptake, acting as iron carriers or auxiliary iron carriers [8, 11]. For instance, catecholamines can markedly promote the growth of multiple bacteria, including *E. coli*, *Pseudomonas aeruginosa* and *Streptococcus pneumoniae* [12–14]. Several pathogenic bacteria encode substrate-binding proteins capable of recognizing catecholamine–iron complexes, with SstD in *S. aureus* [15], PiuA in *Streptococcus pneumoniae*, and PiuA/PirA in *Pseudomonas aeruginosa* [13], responsible for this function.

Another mechanism involves catecholamines acting as signaling molecules. They can activate the bacterial two-component system (TCS), thereby upregulating downstream virulence-related genes [16]. The TCS is a classical bacterial signal-sensing regulatory pathway and detects external stimuli, regulates gene expression, and contributes to environmental adaptation, pathogenesis, and antibiotic resistance [17]. Among these systems, SaeRS represents a key TCS in *S. aureus*. It senses extracellular signals and regulates genes involved in virulence, adhesion, and invasion. Through these functions, it mediates bacterial pathogenesis [4]. In addition, adrenaline and norepinephrine can activate the QseBC system in various Gram-negative bacteria, such as *E*,* coli*. This activation ultimately enhances the aforementioned pathogenic phenotypes [12, 17].

However, current research on interactions between stress hormones—particularly catecholamines—and bacteria has not yet established a rigorous scientific or logical framework. Notably, compared with NE and EPI, DA has received far less attention [18]. This disparity may be associated with host stress responses, as stress markedly elevates in vivo NE and EPI levels, enabling pathogens to perceive these signals, sense host conditions, and initiate invasion [19]. In contrast, systematic investigations into the regulatory role of DA in bacteria remain limited. It is still unclear whether dopamine exerts biologically significant effects on bacteria. Moreover, mechanisms underlying bacterial responses to DA have rarely been reported [20]. Consequently, current understanding in this area remains incomplete and lacks systematic organization.

Accordingly, this study systematically investigated the multifaceted effects of DA on *S. aureus* ATCC 25,923. These effects included bacterial growth, biofilm formation, motility, virulence, and antibiotic resistance phenotypes. Furthermore, the study integrated transcriptomics, proteomics, and metabolomics to explore the underlying regulatory mechanisms. This multi-omics approach enabled a more comprehensive and in-depth analysis of these processes.

## Materials and methods

### Chemicals and strains

Dopamine hydrochloride and chlorpromazine hydrochloride were obtained from Aladdin (Shanghai, China). Phentolamine hydrochloride was purchased from AmBeed (Shanghai, China). All compounds were dissolved in purified water and subsequently sterilized using a 0.22 μm membrane filter. *S. aureus* ATCC 25,923, ATCC 43,300, ATCC 6538, and ATCC 13,565 were acquired from the American Type Culture Collection(ATCC). *S. aureus* ATCC 25,923, a standard clinical reference strain, was selected as the main test strain here [21]. These strains are maintained in our laboratory. *S. aureus* was cultured in Tryptic Soy Broth (TSB). The medium contained 17 g/L tryptic soy protein, 3 g/L soy protein hydrolysate, 5 g/L sodium chloride, 2.5 g/L dipotassium phosphate, and 2.5 g/L glucose. Both ferric chloride (iron(III) chloride) and desferrioxamine (DFO) were purchased from MCE (Shanghai, China) and were used to create iron‑replete and iron‑limited conditions, respectively.

### Measurement of growth curves for *S. aureus* ATCC 25,923

Previous studies have demonstrated that dopamine can promote the growth of certain bacteria [8]. In this study, bacterial growth assays were performed following previously described methods [22]. *S. aureus* ATCC 25,923 was cultured overnight, and the resulting culture was adjusted to an optical density at 600 nm (OD₆₀₀) of 1.0. Subsequently, it was inoculated into TSB medium at a concentration of 1% (v/v). Dopamine was added to obtain final concentrations of 0, 5, 10, 20, 50, 100, 200, 400, and 800 µM. The cultures were incubated at 37 °C with shaking at 180 rpm. Bacterial growth was monitored by measuring OD₆₀₀ at defined time points to generate growth curves. For OD_600_ measurements, an aliquot of the bacterial suspension was placed in a 10 mm path-length cuvette, and the absorbance was measured with a UV-1800PC spectrophotometer (MAPADA, China), using sterile culture medium as the blank.

The generation time was measured over four intervals—1 to 1.5 h, 1.5 to 2 h, 2 to 2.5 h, and 2.5 to 3 h—following a previously described method [23]. In parallel, CFU counts were determined at the 2 h and 3 h time points.

### Measurement of protease activity and biofilm formation

Protease activity was quantified following a previously described method, with minor modifications [24]. Overnight cultures of *S. aureus* were diluted to an OD₆₀₀ of 0.2–0.3. Skim milk agar was prepared using 1/4-strength TSB supplemented with 4% (w/v) skim milk powder and 1.5% (w/v) agar. Dopamine hydrochloride was added before solidification to achieve final concentrations of 0, 5, 10, 20, 50, 100, 200, 400, and 800 µM. Sterile water served as the negative control. A 2 µL aliquot of bacterial suspension was spotted at the center of each agar plate. Plates were incubated at 37 °C for 24 h. Protease activity was assessed by measuring the diameter of the clear zone and the corresponding colony diameter.

Biofilm formation was evaluated using an established method with slight modifications [25]. Overnight cultures of *S. aureus* were inoculated at 1% (v/v) into TSB medium. Dopamine was added to obtain final concentrations of 0, 5, 10, 20, 50, or 100 µM. A volume of 200 µL bacterial suspension was dispensed into each well of a sterile 96-well polystyrene plate (SORFA, Deqing, China). Plates were incubated at 37 °C for 36 h. The suspension was then discarded, and wells were washed three times with 200 µL sterile phosphate-buffered saline (PBS; biosharp, Beijing, China). Cells were fixed with 200 µL of 99% methanol (MACKLIN, Shanghai, China) for 15 min. Methanol was removed, and plates were air-dried. Biofilms were stained with 1% crystal violet (LEAGENE, Anhui, China) for 10 min at 25 °C. Excess dye was gently rinsed off under running water, followed by air-drying. Subsequently, 200 µL of 33% glacial acetic acid (DAMAO, Beijing, China) was added to each well to elute the stain. Plates were incubated at 37 °C for 30 min. Absorbance was measured at 570 nm, and results were normalized to CFU.

### Determination of hemolysin and enterotoxin production

The hemolysis assay was conducted following a previously described method, with necessary modifications [26]. Overnight cultures of *S. aureus* were diluted to an OD₆₀₀ of 0.1. The cultures were then inoculated into TSB medium at 1% (v/v). Dopamine was added to obtain final concentrations of 0, 5, 10, 20, 50, and 100 µM, while sterile water served as the negative control. Cultures were incubated at 37 °C with shaking at 180 rpm for 8 h. Subsequently, 8,000 µL of bacterial suspension was centrifuged at 8,000 × g for 5 min. The supernatant was collected and diluted fivefold with PBS for further analysis. A 5% defibrinated sheep red blood cell suspension (EvaCell, Suzhou, China) was prepared. For the experimental group, 1 mL of red blood cell suspension was mixed with 1 mL of the diluted supernatant in a 2 mL centrifuge tube. For controls, 1 mL of 1% Triton X-100 (BioFroXX, Guangzhou, China) and 1 mL of saline were added as positive and negative controls, respectively. The mixtures were incubated in a 37 °C water bath for 2 h, with gentle mixing every 30 min. Hemolysis plates were prepared by supplementing TSB agar with 10% red blood cell suspension and an appropriate volume of dopamine hydrochloride solution. After cooling and solidification, 1 µL of bacterial dilution was inoculated at the center of each plate. Plates were incubated at 37 °C for 24 h. Hemolytic activity was evaluated using the ratio of the hemolysis zone diameter to the colony diameter.

The determination of enterotoxins was carried out according to a previously reported method, with minor modifications [27]. Overnight cultures of *S. aureus* were diluted to an OD₆₀₀ of 0.1. The cultures were then inoculated into TSB medium at 1% (v/v). After incubation at 37 °C and 180 rpm for 8 h, dopamine hydrochloride was added to obtain final concentrations of 0, 5, 10, 20, 50, and 100 µM. Sterile water was used as the negative control. Incubation was then continued for an additional 8 h. Subsequently, the bacterial suspension was centrifuged. The supernatant was collected and diluted fivefold. Enterotoxin levels were measured using a *S. aureus* Enterotoxin Total Assay Kit (wdwkbio, Beijing, China). The results were normalized to CFU.

### Determination of bacterial motility (swarming and chemotaxis)

Swarming motility assay was performed as previously described with minor modifications [28]. Overnight cultures of S. aureus were diluted to an OD₆₀₀ of 0.1. Sterile filter paper was punched into uniform 4 mm-diameter discs. The discs were autoclaved, dried, and stored for later use. The agar medium contained 10 g/L peptone, 5 g/L glucose, 5 g/L NaCl, and 5 g/L bacterial-grade agar (Aladdin, Shanghai, China). Prior to solidification, dopamine hydrochloride was supplemented to the final indicated concentrations, with sterile water set as the negative control. The prepared filter discs were fully immersed in bacterial suspension and placed in the center of each plate. After incubation at 37 °C for 24 h, the swarming migration diameter was measured.

### Quantitative analysis of dopamine using high performance liquid chromatography–mass spectrometry (HPLC-MS)

The experimental procedure was carried out following a previously described method, with certain modifications [29]. A suspension of *S. aureus* was inoculated into 50 mL of TSB medium at a concentration of 1% (v/v). Cultures were incubated at 37 °C with shaking at 180 rpm for 3 h. Dopamine at 400 µM was then added to the experimental group, while the control group received an equal volume of sterile water. Both groups were incubated for 0.5, 1, and 2 h, respectively. The bacterial suspension was centrifuged at 8,000 × g for 10 min, and the supernatant was collected for analysis. The cell pellet was washed three times and resuspended in 8 mL of PBS. Cells were disrupted using a 40 W ultrasonic disruptor (LD-CP650, Laien, Jining, China) with a 2-second on/4-second off cycle for 5 min. The lysate was centrifuged at 20,000 × g for 20 min, and the supernatant was immediately filtered through a 0.22 μm membrane. A 1.5 mL aliquot of the filtrate was collected for HPLC-MS analysis. To examine the effect of iron on dopamine uptake, the same procedure was followed, except that the initial culture time was reduced to 2 h. Detailed protocols for this section are provided in the HPLC-MS Materials and Methods.docx file.

### Knockout of the *saeS* gene in *S. aureus* ATCC 25,923

The *saeS* gene in *S. aureus* ATCC 25,923 was deleted using a homologous recombination approach mediated by the pBT2 thermosensitive plasmid [30]. The upstream and downstream homologous arms of the *saeS* gene were amplified and fused, then cloned into the pBT2 vector to generate a recombinant knockout plasmid. This plasmid was first transformed into *E. coli* DC10B for demethylation, followed by electroporation into *S. aureus* RN4220 for plasmid amplification. The purified plasmid was subsequently electrotransformed into ATCC 25,923. Positive transformants were selected on chloramphenicol-containing medium at 30 °C. Homologous recombination between the plasmid and the genome was induced at 42 °C. The culture was then passaged at 30 °C without selective pressure to promote plasmid loss. Deletion of the *saeS* gene was confirmed by chloramphenicol sensitivity testing, PCR, and sequencing. Detailed materials and reagents are listed in Table S1.

### Effects of dopamine on the Antibiotic Susceptibility of *S. aureus*

Minimum inhibitory concentrations (MIC) were determined according to the Clinical and Laboratory Standards Institute (CLSI) guidelines to evaluate bacterial antibiotic susceptibility [31]. Polymyxin B Sulfate (PB), Enrofloxacin (ENR), Vancomycin Hydrochloride (VAN), Penicillin G Potassium (PEN), Ceftriaxone Sodium (CRO), Spectinomycin Dihydrochloride (SPT), Levofloxacin (LEV), and Gentamicin Sulfate (GEN) were purchased from AmBeed. Chloramphenicol (CHL), Tetracycline Hydrochloride (TET), Erythromycin (ERY), Streptomycin Sulfate (STR), Kanamycin Sulfate (KAN), Oxacillin (OXA), and Cefoxitin (FOX) were obtained from Solarbio. Two‑fold serial dilutions of each antibiotic were prepared in CAMHB broth, and 100 µL of each dilution was added to the corresponding microplate wells. *S. aureus* suspensions were adjusted to 2 × 10⁶ CFU/mL, and 100 µL of the bacterial suspension was inoculated into each well. For the dopamine treatment group, bacteria were cultured in CAMHB supplemented with 100 µM dopamine. The control group was cultured in CAMHB containing an equal volume of sterile water. All samples were incubated statically at 37 °C for 24 h. Each experiment was performed in six biological replicates to ensure reproducibility. The OD₆₀₀ value was measured using a microplate reader (Model 3020–80359, Singapore) to determine the MIC of each antibiotic. All solvent control groups were set up and processed following the same experimental protocol.

### In vitro adhesion assay

The method was adapted from previous studies with minor modifications [32]. Human immortalized keratinocyte (HaCaT) cells were from National Collection of Authenticated Cell Cultures, Chinese Academy of Sciences (Cat. No. SCSP-5091). HaCaT cells were cultured in Dulbecco’s Modified Eagle’s Medium (DMEM; Gibco, New York, USA) supplemented with 10% fetal bovine serum (FBS; Gibco, New York, USA) in 24-well plates (SORFA, Deqing, China) until reaching a density of 5 × 10³ cells per well. The cells were subsequently centrifuged at 1600 × g for 5 min, washed three times with PBS, and transferred to fresh plates. Preliminary experiments identified 50 µM as the optimal concentration for adhesion. Overnight bacterial cultures were diluted to an OD₆₀₀ of 0.1 and inoculated into TSB medium at 1% (v/v). Dopamine hydrochloride was added to a final concentration of 50 µM, with sterile water serving as the control. Cultures were incubated until OD₆₀₀ reached 1. Following washing and centrifugation, bacterial suspensions were added to the wells at a multiplicity of infection (MOI) of 5:1 and incubated at 37 °C with gentle agitation (25 rpm) for 1 h. After repeated centrifugation and washing to remove non-adherent bacteria, 0.5 mL of 0.1% Triton X-100 was added, and cells were lysed by pipetting. Bacterial counts were determined by serial dilution and subsequent culture on TSB agar plates.

### Invasion and colonization assay of *S. aureus* ATCC 25,923 in mice visceral organs following dopamine treatment

The experimental protocol was adapted from a previous study with minor modifications [33]. Twelve 8-week-old female ICR mice were obtained from Sichuan Lilai Biotechnology Co., Ltd. Mice were maintained in a specific pathogen-free (SPF) facility at 22 ± 2 °C and 50% ± 10% relative humidity under a 12‑h light/dark cycle. They were housed in groups according to experimental group and given ad libitum access to sterilized feed and sterile water. Animals remained in the designated housing area throughout the study. All mice were allowed to acclimatize for 5 days prior to the initiation of experiments. Twelve hours prior to bacterial gavage, the mice received 1.5 mg of dopamine dissolved in 400 µL of PBS, whereas control animals were gavaged with PBS alone. The dopamine dose used in this study was determined in a preliminary experiment, in which doses of 0.5, 1, 1.5, 2, 2.5, and 3 mg were screened. The selection criterion was a significant change in bacterial loads in mouse visceral organs, in the absence of obvious clinical signs or pathologic changes.

Each mouse was subsequently infected with 400 µL of bacterial suspension (2 × 10⁸ CFU/mL) at the predetermined dose. Eight hours post-infection, the animals were euthanized, and the spleen, liver, lungs, kidneys, and heart were aseptically harvested. Serially diluted tissue homogenates were spread onto sheep blood-supplemented TSB agar plates to observe hemolytic activity and colony morphology. Single colonies were randomly picked in batches from plates diluted to 10-2and 10 − 3, cultured in 5 mL TSB medium for 6 h. Colony PCR with universal primers 27 F and 1492R was conducted, and amplicons were separated by agarose gel electrophoresis prior to bidirectional sequencing for strain identification, which eliminated interference from contaminating strains. Bacterial loads were ultimately quantified via colony enumeration. Animals were anesthetized with 2% pentobarbital sodiumat a dose of 35 mg/kg body weight. Euthanasia was performed by exposure to excessive isoflurane inhalation. All animals were confirmed to be anesthetized and unconscious before euthanasia, and dissection was performed only after euthanasia to ensure that animals experienced no pain, in compliance with animal ethics and welfare requirements.

### Scanning electron microscopic morphological observation of *S. aureus*

Sample preparation for scanning electron microscopy (SEM) was performed with minor modifications based on previously reported methods [34]. *S. aureus* was inoculated into 50 mL of TSB liquid medium at 1% (v/v). Dopamine was added to the experimental group to a final concentration of 100 µM, whereas an equal volume of sterile water was used in the control group. Cultures were incubated at 37 °C with shaking at 180 rpm for 3 h. Cells were then collected by centrifugation, washed three times with PBS, and resuspended in electron microscopy fixative, with the bacterial concentration adjusted to 5–10 × 10⁸ CFU/mL to achieve primary fixation. Following fixation, samples were washed three times with 0.1 mol/L phosphate-buffered saline (PBS, pH 7.4) and subsequently post-fixed in 1% osmium tetroxide prepared in PBS for 1–2 h at room temperature in the dark. After an additional rinse with PBS, dehydration was carried out using a graded ethanol series (30%, 50%, 70%, 80%, 90%, 95%, and 100%), combined with isopentanoic acid, with each step lasting 15 min. Dehydrated samples were transferred onto coverslips and dried using a K850 critical point dryer (Quorum, UK). Samples were then mounted onto conductive carbon film double-sided adhesive tape and sputter-coated with a thin layer of gold for 30 s using an MC1000 ion sputter (Hitachi, Japan) to improve electrical conductivity. Finally, morphology was observed and recorded using an SU8100 scanning electron microscope (Hitachi, Japan).

### Transcriptomic, proteomic and metabolomic analyses

Sample preparation was carried out following the protocol provided by Majorbio (Shanghai, China), with minor modifications [35]. The same preparation workflow was applied to both transcriptomic and metabolomic analyses. Preliminary optimization was performed using a concentration gradient ranging from 50 to 800 µM, with increments centered around 50 µM. The optimal dopamine concentration was defined as the lowest concentration that produced the highest ratio, calculated by comparing OD₆₀₀ values at 3 h and 3.5 h (i.e., 30 min after dopamine addition). A 1% (v/v) suspension of *S. aureus* was inoculated into 50 mL of TSB medium and cultured at 37 °C with shaking at 180 rpm until reaching the late logarithmic phase (OD₆₀₀ = 0.80). Dopamine was then immediately added to a final concentration of 400 µM to assess the bacterial response, while the control group received an equal volume of sterile water. After a further 30-minute incubation, cultures were centrifuged and washed, and the resulting pellets were collected for downstream analysis. Each group included three biological replicates, whereas six biological replicates were used for metabolomic analysis. For metabolomics, 50 mL of bacterial culture was subjected to lyophilization prior to analysis. Proteomic sample preparation followed a modified procedure. Dopamine was added at the beginning of cultivation to a final concentration of 100 µM, with sterile water used in the control group. Cultures were incubated under identical conditions for 5 h. Cells were subsequently washed three times with PBS and centrifuged at 8000 × g for 10 min at 4 °C, after which the bacterial pellets were collected for analysis. All raw data were uploaded to the Majorbio cloud platform (https://cloud.majorbio.com) for database searching, data processing, and visualization. The three omics datasets have been deposited in a public repository; detailed information is provided in Supplementary Omics Data.docx.

### Quantitative real-time polymerase chain reaction (RT-qPCR)

RT–qPCR analysis was conducted following previously described methods [36]. The genes *gyrB* and *rpoB* were used as internal reference controls, and the primer sequences for both reference and target genes are provided in the qPCR Primer Sequence Table.xlsx. Total RNA was extracted using a bacterial RNA extraction kit (Yeasen, Shanghai, China) and subsequently reverse transcribed into cDNA with a reverse transcription kit (Yeasen). Quantitative PCR was performed on a Bio-Rad CFX Connect System (Bio-Rad, Singapore) using Hieff UNICON Universal Blue qPCR SYBR Green Master Mix (Yeasen). Given that this study focuses on the SaeRS regulatory pathway, five SaeRS‑associated virulence‑related genes *(saeR*, *hlgB*, *hlgC*, *emp*, and *sbi*) were prioritized from the pool of differentially expressed genes that met the criteria of |FC| > 1.5 and FDR < 0.05. The remaining ten genes were equally divided into up‑regulated and down‑regulated groups and randomly selected from the same pool of qualifying DEGs using the “RAND()” function in Microsoft Excel (2021, Version 16.54), in order to minimize selection bias.

#### Statistical analysis

Unless otherwise specified or in preliminary experiments, all assays were performed independently in triplicate, with three parallel samples included in each group. Data presented in the figures are expressed as the standard error of the mean (SEM) derived from three independent experiments. Statistical analyses were conducted using t-tests in Origin 2024 software, with *p* < 0.05 considered statistically significant. Significance levels were indicated as *p* < 0.05 (*), *p* < 0.01 (**), and *p* < 0.001 (***).

## Results

### *S. aureus* ATCC 25,923 can uptake dopamine molecules

**Fig. 1:**
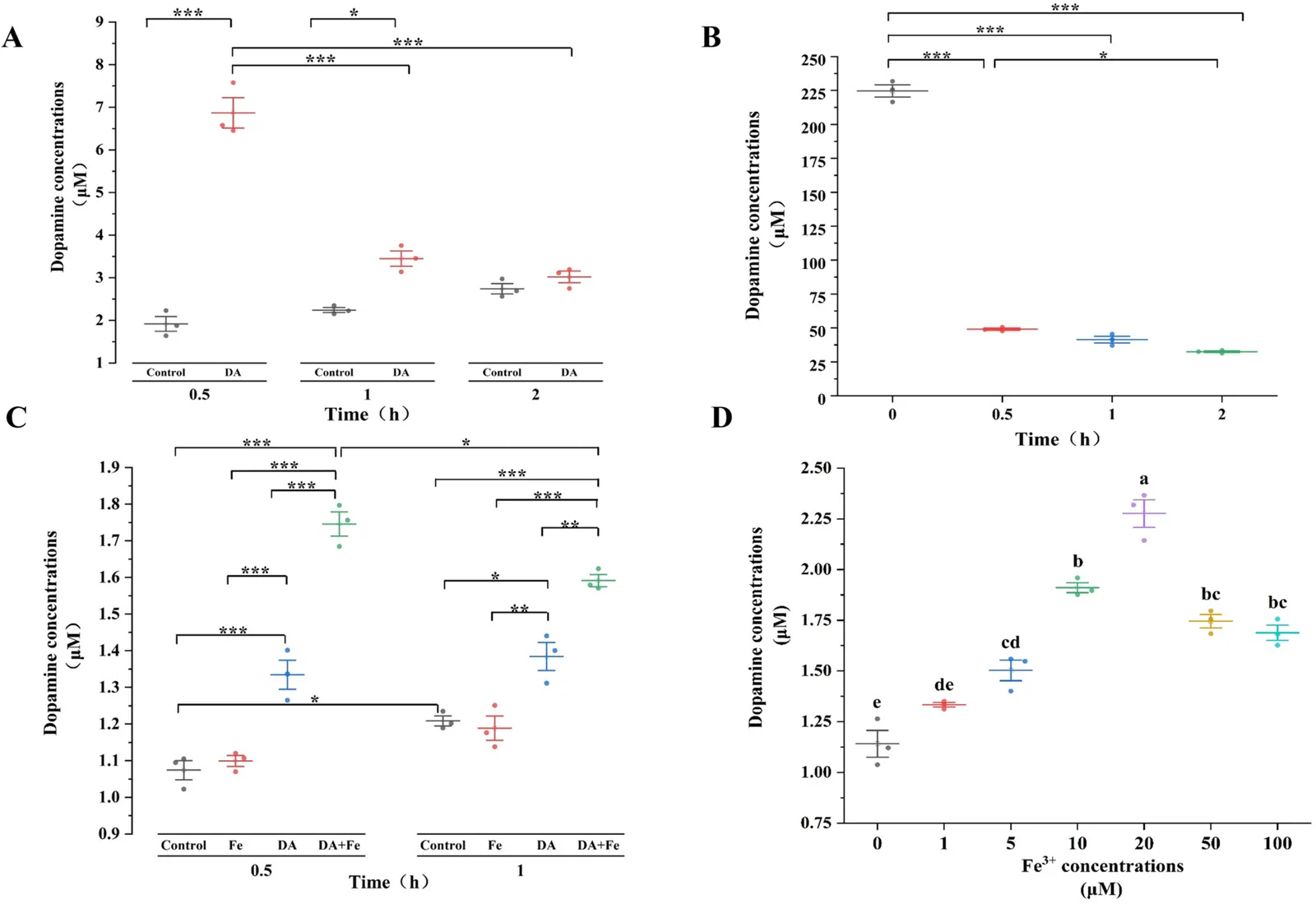
Determination of dopamine concentrations by HPLC–MS. “Control” denotes the group receiving an equal volume of sterile water only, whereas “DA” “Fe” and “DA + Fe” indicate the respective treatment groups. Owing to the growth-promoting effect, all data were normalized to CFU. **A** Intracellular dopamine concentrations in bacteria at different time points. **B** Dopamine concentrations in the supernatant at different time points; 0 h represents measurements taken immediately after mixing following dopamine addition. **C** Effect of exogenous iron on bacterial uptake of dopamine. Dopamine and FeCl₃ were both applied at a final concentration of 50 µM. **D** Exogenous iron promotes dopamine uptake by *S. aureus*. Different lowercase letters indicate statistically significant differences between groups (*p* < 0.01); groups sharing the same letter do not differ significantly. This figure and the corresponding description have been added to the main text to further validate the role of iron in facilitating this process

Relative to the control group, dopamine supplementation led to a significant increase in intracellular dopamine levels in *S. aureus* at 0.5 and 1 h, followed by a gradual decline as incubation progressed (Fig. 1A). When 400 µM dopamine was added to the culture, the measured concentration was approximately 225 µM. In parallel, dopamine levels in the supernatant decreased markedly over time (Fig. 1B). As illustrated in Fig. 1C, co-treatment with iron and dopamine resulted in a significantly higher intracellular dopamine concentration compared with either treatment alone. Collectively, these findings suggest that dopamine can be internalized by bacterial cells, that this process is enhanced by iron, and that it may involve a metabolic pathway. To further characterize this iron‑enhanced uptake, we examined the effect of increasing Fe³⁺ concentrations. As shown in Fig. 1D, exogenous supplementation with Fe³⁺ at 5, 10, 20, 50, and 100 µM significantly promoted dopamine uptake by *S. aureus* compared with the control, with the maximal effect observed at 20 µM. Although dopamine uptake increased in a concentration‑dependent manner, the efficiency declined slightly at concentrations above 20 µM.

### Transcriptomic analysis

**Fig. 2:**
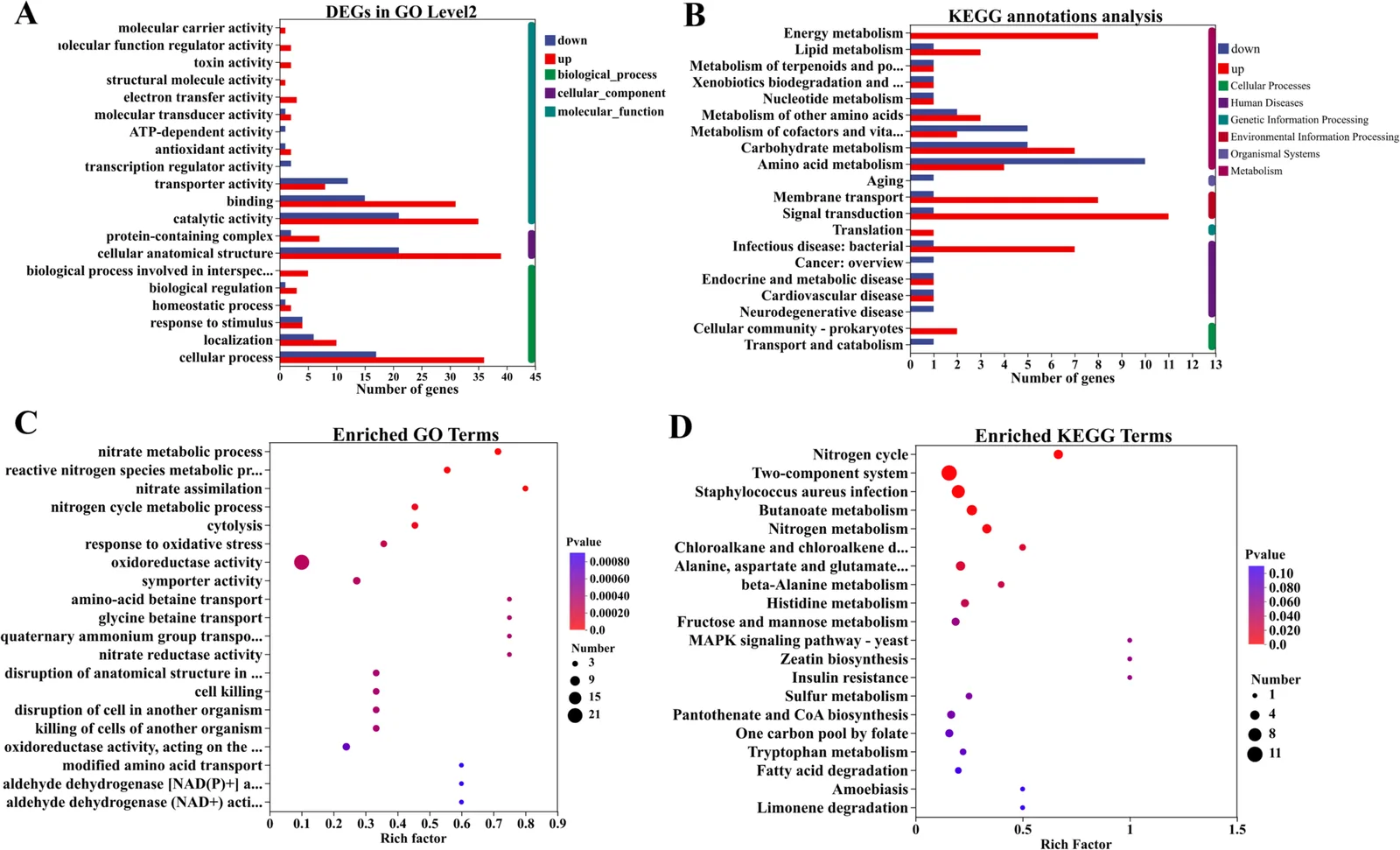
Annotation and enrichment analysis of DEGs. **A** GO annotation; **B** KEGG annotation; **C** GO enrichment analysis; **D** KEGG enrichment analysis. GO/KEGG enriched terms are arranged from top to bottom in descending order of significance (i.e., significance decreases from top to bottom)

Using a threshold of log₂ fold change (FC) ≥ 1.5 and a false discovery rate (FDR) ≤ 0.05, transcriptomic analysis identified 117 differentially expressed genes (DEGs) in dopamine-treated *S. aureus* ATCC 25,923 compared with the control group, including 71 upregulated and 46 downregulated genes (DEGs Details Table.xlsx). To further characterize the bacterial response to dopamine, DEGs were subjected to Gene Ontology (GO) and Kyoto Encyclopedia of Genes and Genomes (KEGG) functional annotation. Across the three GO categories—biological processes, cellular components, and molecular functions—the number of upregulated genes markedly exceeded that of downregulated genes, particularly in functional groups associated with regulation, toxins, and catalytic activity (Fig. 2A). KEGG pathway analysis showed that dopamine treatment generally enhanced core metabolic pathways in *S. aureus*, including energy metabolism, carbohydrate metabolism, and lipid metabolism. In addition, pathways related to signal transduction and virulence were activated, whereas amino acid metabolism exhibited a downregulated trend (Fig. 2B). Overall, functional annotation of the DEGs suggests that dopamine promotes broad physiological activities in *S. aureus*.

GO enrichment analysis showed that DEGs were predominantly associated with pathways involved in nitrogen metabolism, redox processes, oxidative stress response, and virulence (Fig. 2C). Consistently, KEGG enrichment analysis indicated significant enrichment of DEGs in nitrogen metabolism, two-component signaling systems, *S. aureus* infection, and amino acid metabolism pathways (Fig. 2D). These findings are in agreement with the GO results, collectively indicating that dopamine modulates nitrogen utilization, signal transduction, and virulence-related processes in *S. aureus*, while also affecting its core metabolic networks. Notably, 11 genes associated with the two-component system were identified within the signaling pathways, suggesting a potential role for dopamine in bacterial sensing and regulatory mechanisms.

DEGs were annotated using the Comprehensive Antimicrobial Resistance Database (CARD), and detailed results are presented in the CARD Annotation Details Table (Fig. S1B). The vancomycin resistance regulator gene *vanR*, together with genes associated with tetracycline and macrolide resistance, exhibited increased expression. Most genes within the glycopeptide antibiotic resistance cluster showed a similar upregulation pattern. By comparison, genes related to peptidoglycan-targeting antibiotics were downregulated. A comparable decrease was observed for the majority of genes in the ATP-binding cassette (ABC) transporter-associated efflux pump cluster. Overall, these findings suggest that dopamine modulates the expression of antibiotic resistance-related genes in *S. aureus*.

### RT-qPCR analysis

Functional annotations and expression profiles of all genes described in this section are provided in the file “Description of qPCR Gene Functions.xlsx.” Fifteen genes were randomly selected for RT–qPCR validation. The RT–qPCR results were consistent with the transcriptomic data, thereby supporting the reliability of the transcriptomic analysis.

A substantial number of genes associated with the SaeRS two-component regulatory system were identified among the DEGs. In particular, the core regulatory genes *saeS* (FC = 1.89) and *saeR* (FC = 2.03), together with their direct downstream virulence-associated targets (*efb*, *hlgA*, *hlgB*, and *hlgC*), were significantly upregulated. To further explore the underlying mechanism, the *saeS* gene was deleted in the wild-type (WT) *S. aureus* ATCC 25,923 strain, generating the △*S* mutant (Fig. S2). Recent studies have suggested that dopamine supplementation promotes bacterial iron uptake, a finding also supported by the results shown in Fig. 1. However, among the DEGs identified in this study, only *isdC* (FC = 5.11) and *fur* (FC = 3.78) were associated with iron transport, and neither exhibited statistically significant changes. Similarly, several virulence-related genes, including the enterotoxin-associated genes *seg* (FC = 1.76), *seu* (FC = 1.87), and *sei* (FC = 2.01), displayed increased expression without reaching statistical significance (*p* ≥ 0.5). In addition, other genes linked to the SaeRS regulatory system, including certain downstream targets, did not show significant upregulation (FC ≤ 1.5 or *p* ≥ 0.5). Based on these observations, dopamine concentration was subsequently adjusted, and RT–qPCR analysis was performed to further examine the expression of the corresponding genes.

Notably, as shown in Fig. S3, comprehensive RT–qPCR analysis demonstrated that *saeRS* expression was markedly higher than that observed in the transcriptomic data. Concurrently, multiple genes involved in iron transport and metabolism were significantly upregulated, including *isdCEFG* (Isd heme–iron uptake system), *sirABC* and *sbnABCD* (staphylococcal iron carrier B synthesis and secretion systems), *efeO* (iron-binding protein), and *feoB* (ferrous iron transporter). Although fur expression decreased by 2.57-fold, it remained at a relatively high expression level. At this dopamine concentration, enterotoxin gene clusters (*seg*, *sen*, *seu*, and *sei*) were significantly upregulated as a group. In addition, multiple virulence-associated genes—including *capA* (capsular polysaccharide synthase), coa (coagulase), *fnbA* and *fnbB* (fibrinogen-binding proteins), *emp* (extracellular matrix-binding protein), *fib* (fibrinogen-binding protein), *hlgABC* (γ-hemolysin), *hld* (δ-hemolysin), *hyl* (α-hemolysin, this discrepancy results from incorrect annotation of the reference genome; the correct gene is actually *hla*), *ssl* (*staphylococcal* superantigen-like proteins), and *sbi* (immune evasion protein)—as well as other downstream targets of the *saeRS* system, also showed significant upregulation. A similar trend was observed for the *agrABC* quorum-sensing system. Taken together, these results indicate that dopamine enhances iron acquisition and metabolic activity in *S. aureus*, activates the SaeRS regulatory system, and promotes the expression of virulence factors in a concentration-dependent manner.

As shown in Fig. S4, deletion of the *saeS* gene resulted in an overall downregulation of genes downstream of the SaeRS system compared with the WT strain. Following treatment with the same concentration of dopamine, expression levels exhibited minor fluctuations but remained significantly lower than those observed in the dopamine-treated WT group. Interestingly, enterotoxin genes not directly regulated by the SaeRS system displayed a similar trend. In contrast, the *agr* quorum-sensing system appeared unaffected. These findings suggest that *S. aureus* primarily senses dopamine through the SaeRS system, which subsequently modulates the expression of downstream genes.

**Fig. 3:**
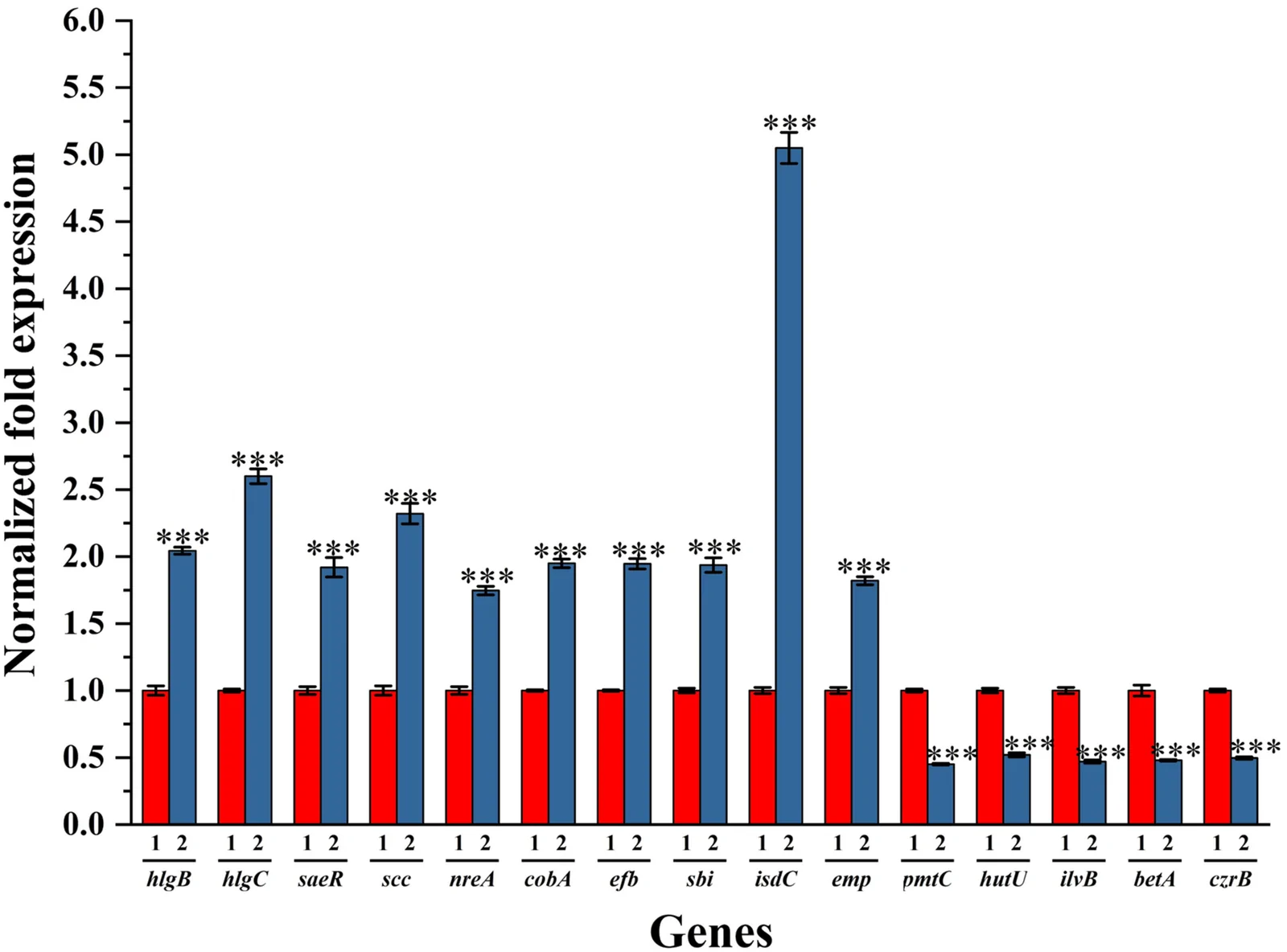
Results of RT-qPCR analysis. 1, Control group treated with an equal volume of sterile water; 2, Treatment group supplemented with 400 µM dopamine

### Proteomic analysis

**Fig. 4:**
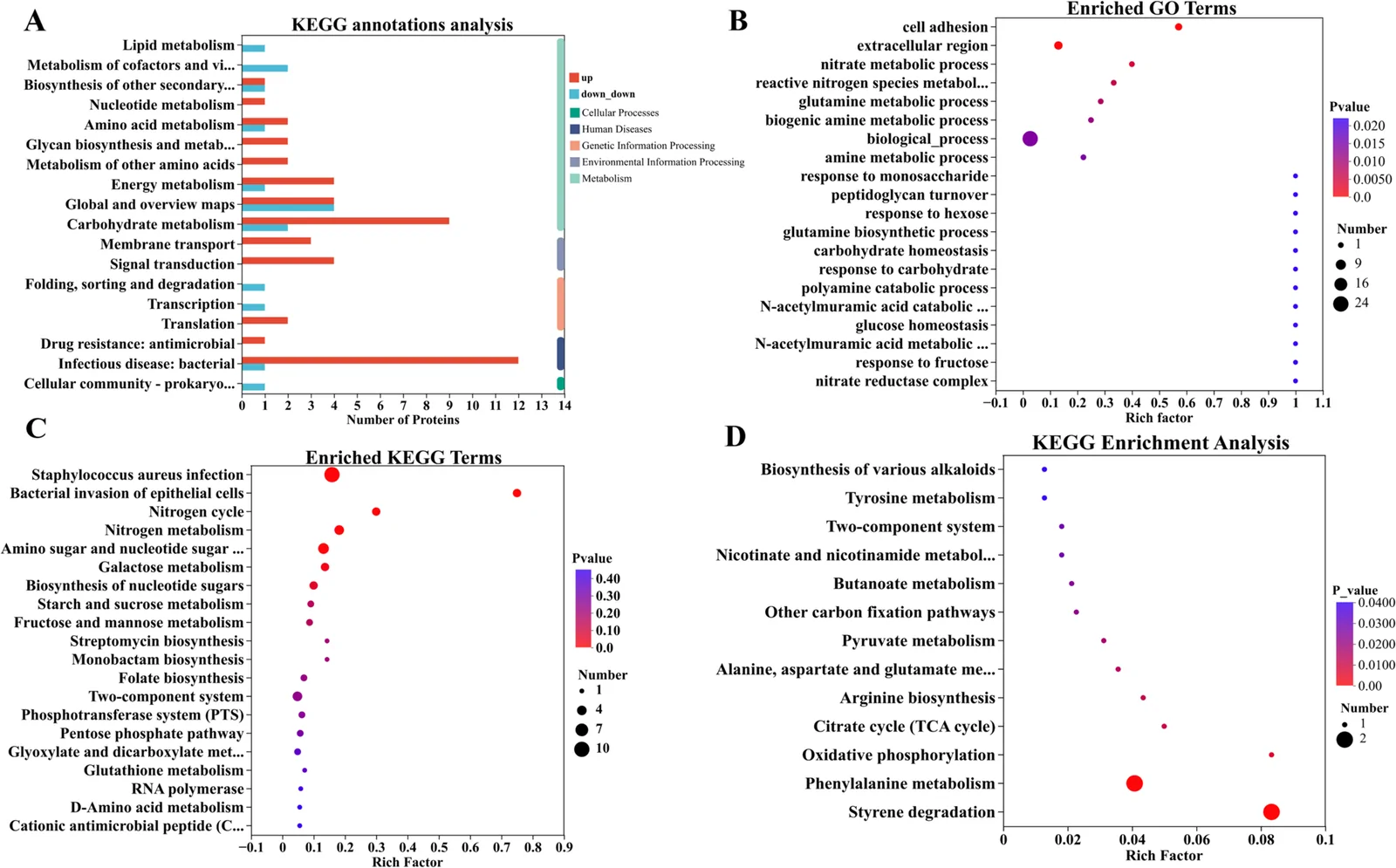
Results of proteomic and metabolomic analyses. **A** KEGG pathway annotation of DPGs; **B** GO enrichment analysis of DPGs; **C** KEGG enrichment analysis of DPGs; **D** KEGG enrichment analysis of DEMs

Using thresholds of FC ≥ 1.15, FC ≤ 0.87, and FDR ≤ 0.05, proteomic analysis comparing the dopamine-treated group with the sterile water-treated control identified 91 differentially expressed proteins (DEPs), including 49 upregulated and 42 downregulated proteins (DEPs Details Table.xlsx). KEGG annotation indicated that upregulated proteins predominated and were mainly enriched in pathways related to bacterial infection, carbohydrate metabolism, and energy metabolism. In parallel, signaling pathways were also significantly enriched, suggesting coordinated activation of virulence-associated functions and core metabolic networks. In contrast, downregulated proteins were distributed across a limited number of pathways and did not show a clear enrichment pattern (Fig. 4A). Among the upregulated proteins, several were associated with host interaction and virulence. ClfA (FC = 1.63) and ClfB (FC = 1.30), which mediate binding to host fibrinogen and promote adhesion and aggregation, showed increased expression. FnbB (FC = 2.04), involved in adhesion to the extracellular matrix and facilitating colonization and invasion, was also upregulated. In addition, Spa (FC = 1.96), associated with immune evasion, and IsdA (FC = 1.44), a key component of the iron acquisition system, exhibited elevated expression levels. GO enrichment analysis further highlighted that DEPs were mainly associated with cell adhesion, extracellular components (largely reflecting virulence factor expression), and nitrogen metabolism-related processes (Fig. 4C). Consistent with these findings, KEGG enrichment analysis showed that the *S. aureus* infection pathway displayed the highest enrichment and strongest statistical significance. Pathways related to bacterial invasion of epithelial cells, nitrogen metabolism, and carbohydrate metabolism were also significantly enriched. Notably, enrichment was also observed in two-component regulatory systems and antibiotic resistance pathways. Collectively, these results indicate that dopamine enhances virulence factor expression in *S. aureus* while simultaneously promoting metabolic activity. The enrichment of nitrogen metabolism-related pathways further suggests that dopamine may contribute to nitrogen utilization in *S. aureus*.

### Metabolomic analysis

Metabolomic profiling was conducted to compare the dopamine-treated (DA) group with the untreated control. Using thresholds of *p* < 0.05, Variable Importance in Projection (VIP) > 1, and FC > 1.05 or FC < 0.93, and excluding dopamine-related compounds, a total of 15 differentially expressed metabolites (DEMs) were identified, including 11 upregulated and 4 downregulated metabolites (DEMs Details Table.xlsx). Among the significantly upregulated metabolites, Pa (Pgf1Alpha/19:2(10Z,13Z)), Lpg (17:0), and tuberculostearic acid are glycerophospholipids containing polyunsaturated fatty acid chains or branched-chain saturated fatty acids, which are involved in bacterial membrane biosynthesis. This enrichment may be associated with the observed promotion of biofilm formation by dopamine. In addition, phenylacetic acid, a metabolite of phenylalanine, and 2-mercaptoethanol, a reducing agent involved in oxidative stress mitigation, were also upregulated. KEGG pathway enrichment analysis further indicated that DEMs were primarily enriched in phenylalanine metabolism, two-component systems, and energy metabolism pathways, including the TCA cycle and oxidative phosphorylation.

### Dopamine promotes the growth of *S. aureus* ATCC 25,923

**Fig. 5:**
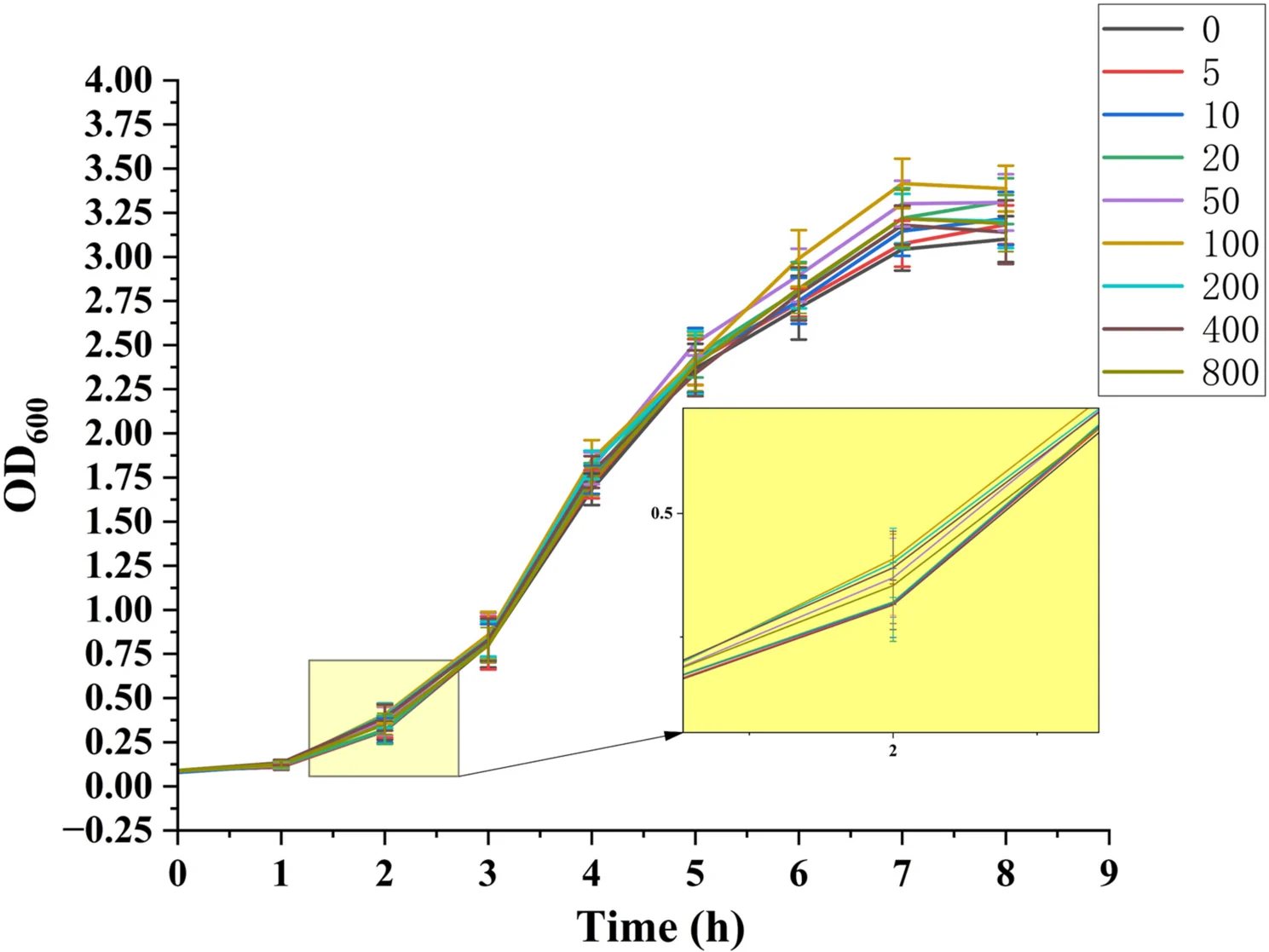
Effects of dopamine on the growth of *S. aureus* ATCC 25,923. Numbers 0–800 represent dopamine concentrations (µM); group 0 served as the control, treated with an equal volume of sterile water

Relative to the control, exogenous dopamine supplementation enhanced the growth rate of *S. aureus* ATCC 25,923. The most pronounced effect was observed at 100 µM after 2 h of incubation, where the growth rate increased by 29.52% (Fig. 5). When FeCl₃ was co-incubated with dopamine, the growth-promoting effect was further amplified compared with the control. The magnitude of this effect followed the order: FeCl₃ + DA > FeCl₃ > DA (Fig. S5A). Addition of the iron chelator deferoxamine (DFO) markedly inhibited bacterial growth. Notably, supplementation with dopamine at the same concentration restored growth to levels comparable to the control (Fig. S5B). For the △*S* mutant strain, growth patterns were similar to those of the wild-type (WT) under dopamine treatment at concentrations of 0, 50, 100, and 200 µM, with no appreciable differences observed (Fig. S5C).

100 µM dopamine markedly shortened the generation time of *S. aureus* and promoted proliferation only during the 1.0–1.5 h interval; at later time intervals, generation times did not differ appreciably between groups (Fig. S6A). CFU counts at 2 h and 3 h showed that the viable cell number in the dopamine‑treated group was significantly higher than that in the control group (Fig. S6B).

### Dopamine enhances the virulence of *S. aureus* ATCC 25,923

**Fig. 6:**
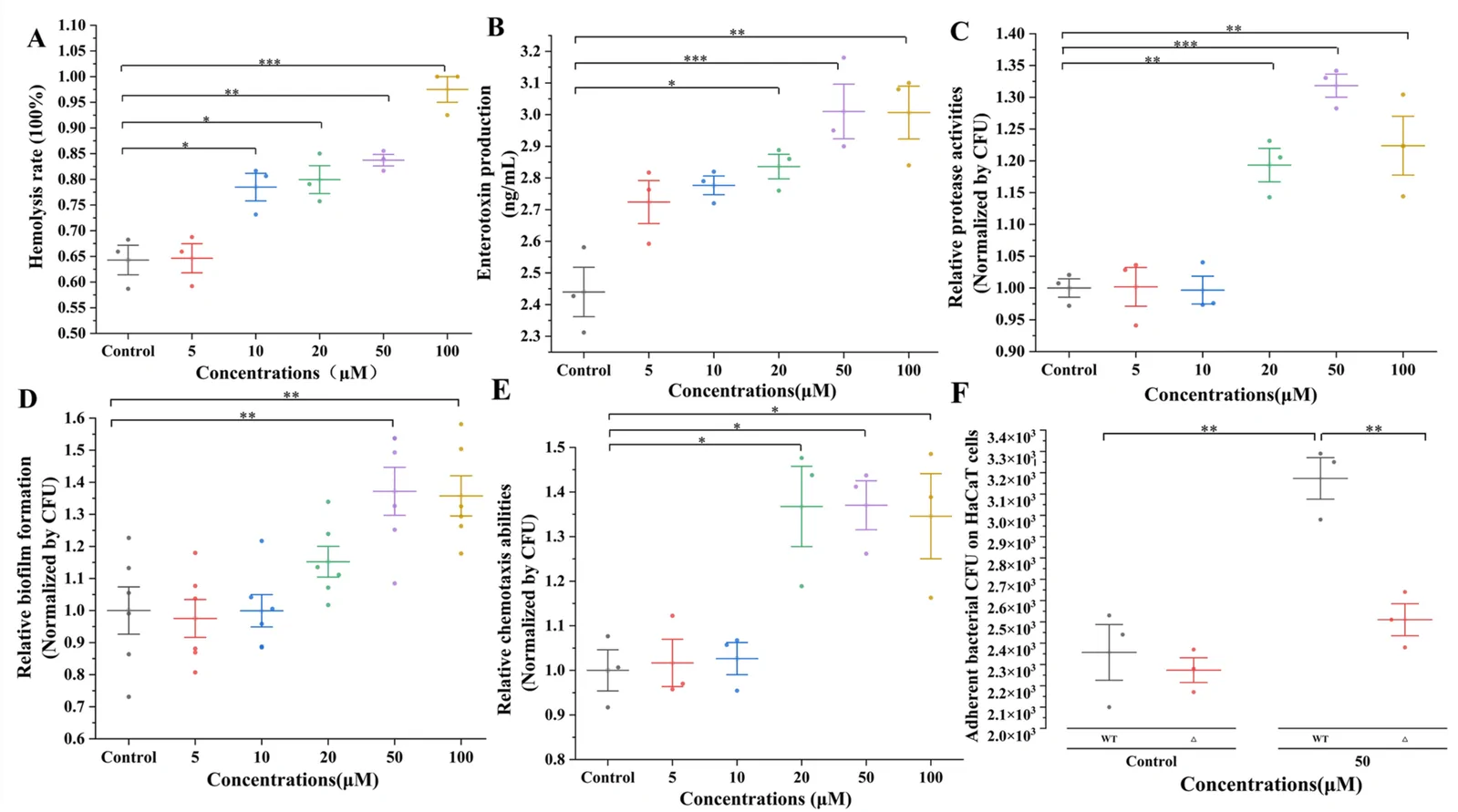
Effects of dopamine on virulence- and colonization-related phenotypes in *S. aureus*. Sterile water was used as the control. **A** Hemolysis rate (5-fold dilution); **B** Enterotoxin production (5-fold dilution); **C** Protease activity; **D** Biofilm formation; **E** Chemotaxis; **F** Adhesion. The symbol △ indicates the *saeS*-knockout strain derived from the WT strain

As shown in Fig. S7 and Fig. S8A, dopamine treatment significantly enhanced the total hemolytic activity of *S. aureus*. It should be noted that this plate‑based assay measures overall hemolysis and cannot distinguish among the individual hemolytic phenotypes (e.g., α‑, γ‑, δ‑hemolysin, or PSMs); the specific contribution of each hemolytic factor can only be inferred from gene expression data. The hemolysis rate in the control group was 64.29%, whereas supplementation with 10, 20, 50, and 100 µM dopamine increased the hemolysis rate by 22.09%, 34.36%, 30.25%, and 51.67%, respectively (Fig. 6A). A similar trend was observed for enterotoxin production. Following dopamine treatment, enterotoxin levels increased significantly, with elevations of 16.28%, 23.53%, and 23.39% at concentrations of 20, 50, and 100 µM, respectively (Fig. 6B).

### Dopamine enhances protease activity and motility of *S. aureus* ATCC 25,923

As illustrated by the plate images in Fig. 8C and Fig. S10B, dopamine treatment significantly enhanced the protease activity of *S. aureus* ATCC 25,923. At concentrations of 20, 50, and 100 µM, protease activity increased by 19.33%, 31.83%, and 22.39%, respectively. Chemotactic ability exhibited a similar pattern, with increases of 36.76%, 37.03%, and 34.56% at the corresponding dopamine concentrations (Fig. 6E). Consistent with these findings, plate assays shown in Fig. S8C and Fig. S10 demonstrated that clustering rates were elevated following dopamine treatment, with increases of 28.06% and 39.57% at 20 and 50 µM, respectively.

### Dopamine enhances biofilm formation and adhesion ability of *S. aureus* ATCC 25,923

Compared with the control group, dopamine supplementation significantly promoted biofilm formation in *S. aureus* ATCC 25,923. At concentrations of 50 and 100 µM, biofilm formation increased by 37.18% and 35.75%, respectively (Fig. 6D). In vitro adhesion assays further showed that exogenous dopamine enhanced bacterial adhesion to HaCaT cells. At 50 µM, adhesion increased by 34.65% compared with the control group (Fig. 6F).

### Dopamine enhances the invasion and colonization capabilities of *S. aureus* ATCC 25,923 in Mice

**Fig. 7:**
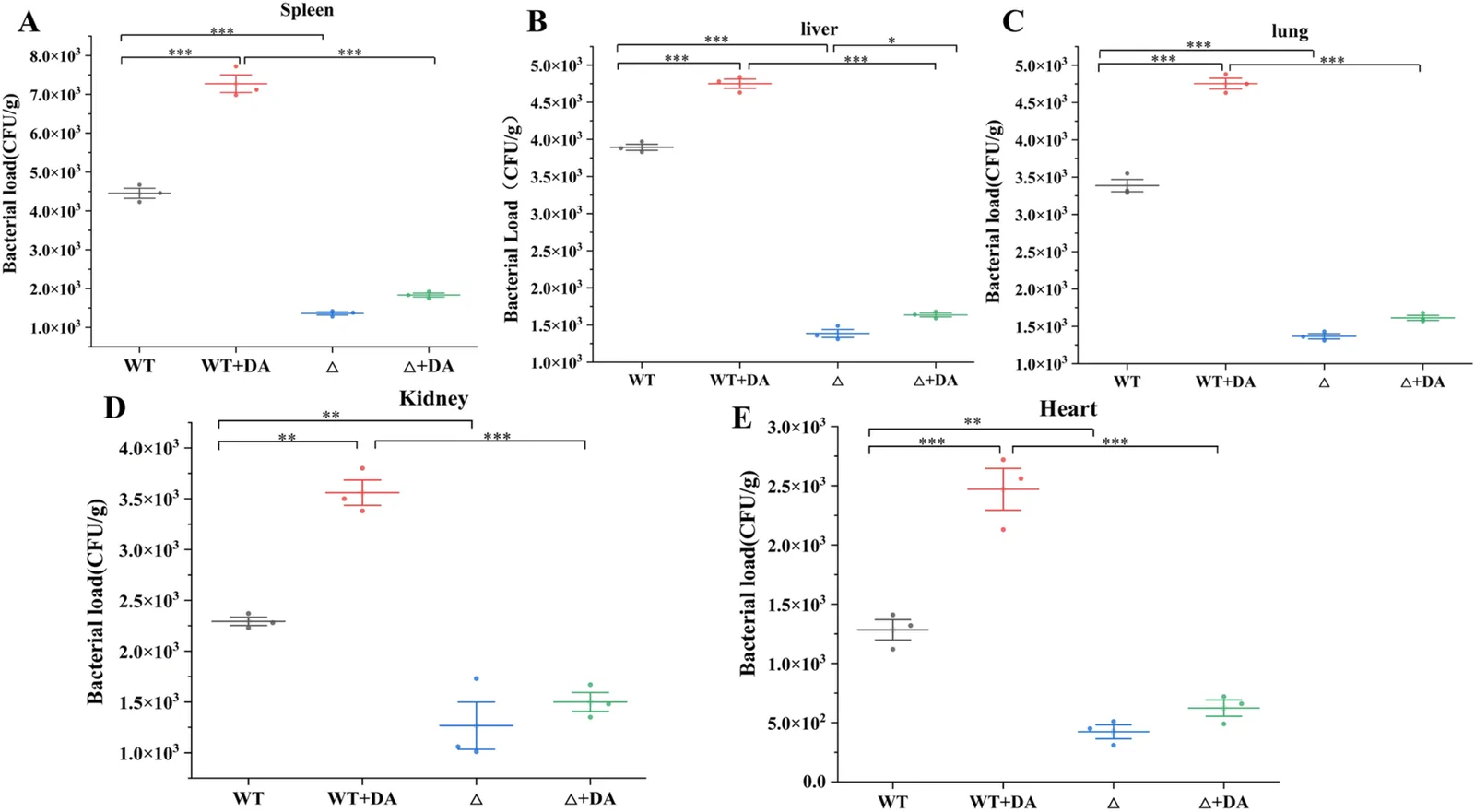
Bacterial loads in visceral tissues of mice with or without dopamine pretreatment. **A** Spleen; **B** Liver; **C** Lung; **D** Kidney; **E** Heart. DA represents dopamine pretreatment. The WT and Δ*saeS* groups were treated with an equal volume of PBS, and the DA group was treated with 100 µM dopamine

As shown in Fig. 7, bacterial loads in the control group varied across visceral tissues, ranging from 1.0 to 8.0 × 10³ CFU/g. In descending order, the distribution was spleen, liver, lung, kidney, and heart. This pattern indicates that systemic infection by *S. aureus* in mice exhibits tissue-specific distribution, with significantly higher bacterial burdens in the spleen and liver than in other organs, suggesting a preferential colonization of reticuloendothelial system-associated tissues. Following dopamine pretreatment, bacterial loads increased markedly in all examined tissues. In contrast, the △*S* mutant lacking the *saeS* gene showed a pronounced reduction in invasion and colonization capacity compared with the WT strain, with highly significant differences. These findings further support a critical role for *saeS* in systemic infection processes, including invasion, virulence, and immune evasion. When dopamine was applied to the △*S* strain, bacterial loads remained substantially lower than those observed in the dopamine-treated WT group, with highly significant differences. Dopamine-treated △*S* group with the PBS-treated control, only a slight increase in bacterial load was detected, reaching statistical significance in the liver but not in other tissues. Collectively, these results suggest that dopamine may enhance systemic dissemination of *S. aureus* ATCC 25,923 by modulating downstream virulence gene expression via the SaeRS regulatory system, thereby promoting invasion and colonization during infection.

### Scanning electron microscope observation of the morphology of *S. aureus* ATCC25923

**Fig. 8:**
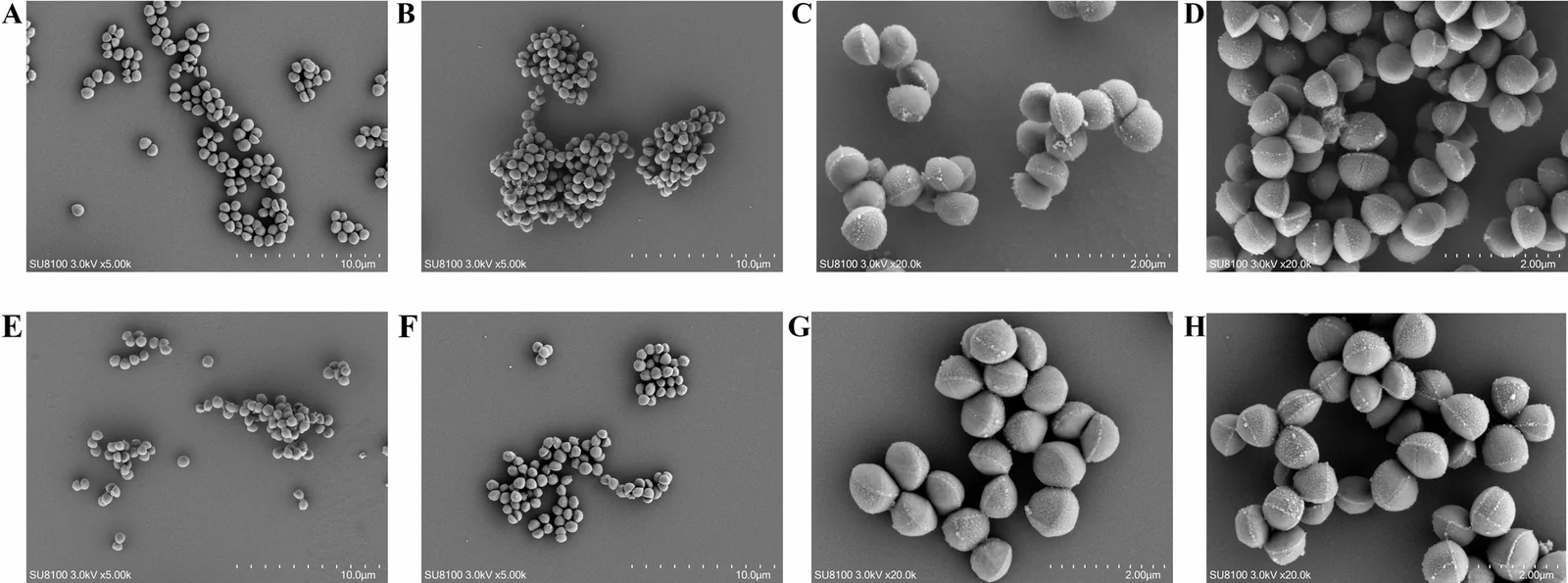
Morphological observation of *S. aureus* under scanning electron microscopy. The scale bars in the bottom right corner represent 2 μm and 10 μm, respectively. Panels A–B–E–F and C–D–G–H show observations at two different magnifications. A–D correspond to the WT strain: A and C are the control groups treated with an equal volume of sterile water, while B and D are the treatment groups supplemented with 100 µM dopamine. E–H correspond to the Δ*saeS* strain: E and F are the control groups treated with an equal volume of sterile water, while G and H are the treatment groups supplemented with 100 µM dopamine

SEM revealed that *S. aureus* cells in the WT control group were predominantly dispersed or formed small aggregates (Fig. 8A, C). In contrast, dopamine-treated WT cells exhibited more compact, grape-like clusters with tighter intercellular adhesion. Cell surfaces appeared denser and were surrounded by increased extracellular matrix, forming continuous biofilm-like structures (Fig. 8B, D). These observations indicate that dopamine enhances bacterial adhesion and aggregation. A similar trend was observed in the △*S* mutant compared with the WT strain, suggesting that deletion of the *saeS* gene affects biofilm formation and adhesion. Notably, dopamine treatment of the △*S* strain also resulted in increased aggregation and extracellular matrix production relative to its untreated control, accompanied by a denser cell surface. However, these changes were less pronounced than those observed in the dopamine-treated WT group. These findings suggest that, in addition to the SaeRS system, dopamine may influence the morphology and surface characteristics of *S. aureus* through additional regulatory pathways. Concurrently, SEM observations showed that under 100 µM dopamine treatment, the cell size of *S. aureus* remained comparable to that of the control group. Together with the growth curves, generation time data, and CFU counts, these findings indicate that dopamine accelerates bacterial replication, thereby increasing the overall growth rate.

### Dopamine increases antimicrobial resistance in *S. aureus*

**Fig. 9:**
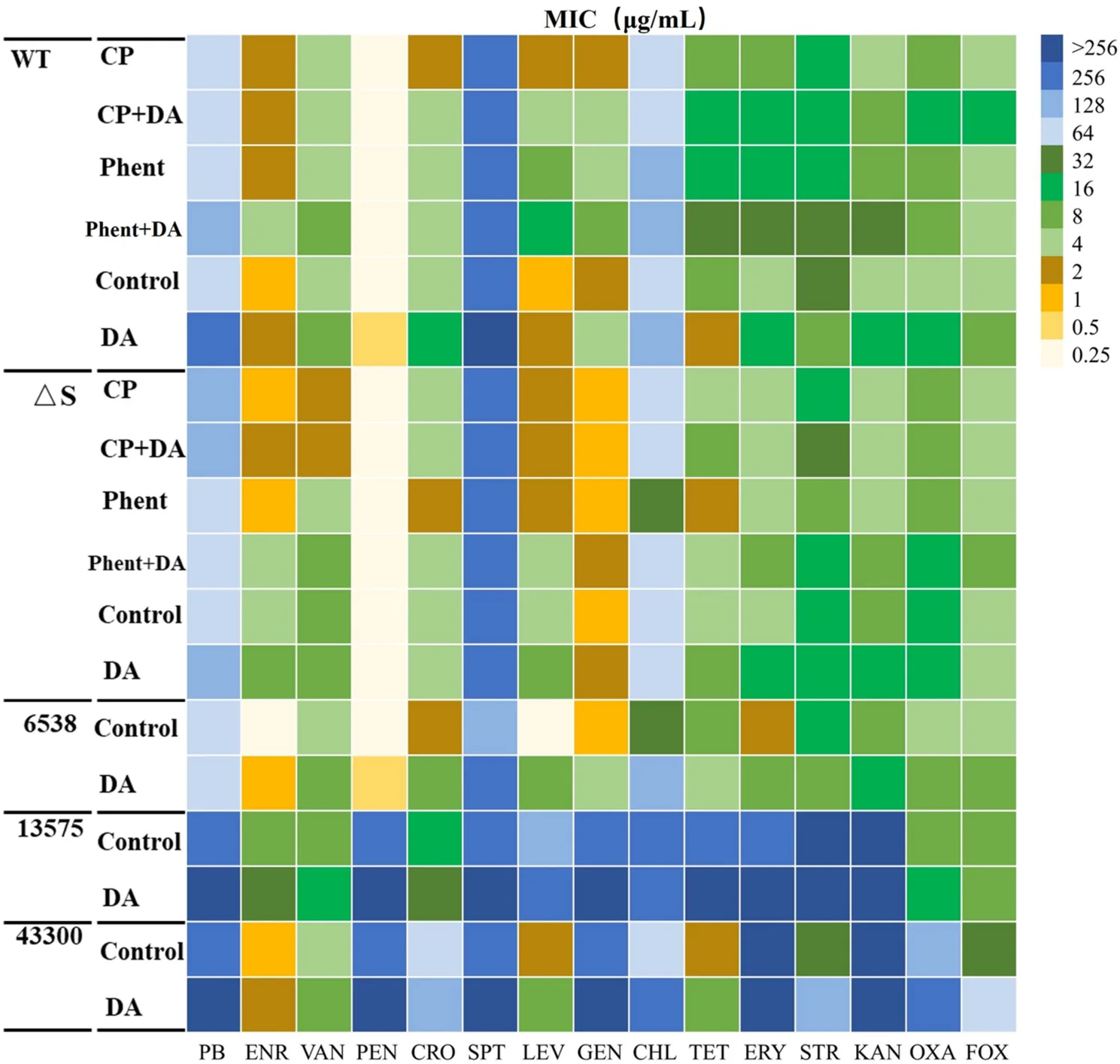
Effects of dopamine on the minimum inhibitory concentration (MIC) of four *S. aureus* strains against 15 antibiotics. Control group was treated with sterile water only. The final concentrations of exogenous DA, CP and Phent were all 100 µM. Polymyxin B Sulfate (PB); Enrofloxacin (ENR); Vancomycin Hydrochloride (VAN); Penicillin G Potassium (PEN); Ceftriaxone Sodium (CRO); Spectinomycin Dihydrochloride (SPT); Levofloxacin (LEV); Gentamicin Sulfate (GEN); Chloramphenicol (CHL); Tetracycline Hydrochloride (TET); Erythromycin (ERY); Streptomycin Sulfate (STR); Kanamycin Sulfate (KAN); Oxacillin (OXA); Cefoxitin (FOX)

To assess the impact of dopamine on antibiotic susceptibility, 15 antibiotics were tested across four *S. aureus* strains. As shown in Fig. 9, the dopamine-treated (DA) group generally exhibited higher MIC values compared with the control group, indicating reduced antibiotic susceptibility. For *S. aureus* ATCC 25,923, removal of dopamine led to a decrease in MIC values for tetracycline hydrochloride and streptomycin sulfate, from 8 to 2 µg/mL and from 32 to 8 µg/mL, respectively. A similar trend was observed in another set of measurements, with values decreasing from 8 to 4 µg/mL and from 16 to 8 µg/mL for these two antibiotics. In addition, for other strains, including ATCC 13,575 and ATCC 43,300, MIC values increased for most of the tested antibiotics. Overall, dopamine treatment resulted in elevated MIC values across strains, with the majority showing 2- to 4-fold increases, while only a few remained unchanged or increased by a single fold. Notably, the extent of these changes varied among strains. In strain ATCC 25,923, deletion of the *saeS* gene attenuated or abolished the dopamine-induced increase in MIC.

Furthermore, the non-selective α1/α2-adrenergic receptor blocker phentolamine hydrochloride and the dopamine receptor antagonist chlorpromazine effectively reduced the dopamine-associated increase in MIC. However, this inhibitory effect was largely lost in strains lacking the *saeS* gene.

### *S. aureus* may sense dopamine mainly via the SaeRS system and upregulate downstream genes

Dopamine significantly enhances multiple phenotypic traits of *S. aureus* ATCC 25,923, including biofilm formation, growth, invasiveness, virulence, protease activity, antibiotic resistance, and motility. To further elucidate the underlying mechanisms, the effects of dopamine were examined in the *saeS* knockout strain. As shown in Figs. 6E and 7, and Fig. S10, deletion of *saeS* led to a reduction in nearly all phenotype-associated activities in *S. aureus* ATCC 25,923. Although dopamine supplementation still promoted these phenotypes in the knockout strain, the magnitude of enhancement was significantly lower than that observed in the WT strain, with statistically significant differences across multiple concentrations. For instance, at 20 and 50 µM dopamine, differences in clustering and hemolytic activity were highly significant, while at 50 µM, biofilm formation, enterotoxin production, and adhesion also showed highly significant differences.

SaeS kinase activity has been reported to be critical for pathogen survival [37], a finding further supported by the in vivo mouse infection results in this study (Fig. 7). These observations also underscore the role of dopamine in promoting infection, invasion, and colonization of *S. aureus* in the host [33]. Interestingly, the phenotypic effects were more pronounced at lower dopamine concentrations. Integration of omics data, RT–qPCR results, and phenotypic analyses indicates that the SaeRS system serves as the primary regulatory pathway mediating dopamine sensing and downstream responses in S. aureus. However, even in the absence of *saeS* and with partial disruption of the SaeRS system, the bacteria retain a residual capacity to respond to dopamine, suggesting the involvement of additional regulatory pathways that contribute to the modulation and enhancement of these phenotypes.

## Discussion

In this study, the expression of genes associated with phenotypes and underlying mechanisms showed a high degree of correspondence. These included hemolysin-related genes (*hyl*, *hld*, and *hlgABC*), enterotoxin genes (*seg*, *sen*, *seu*, *sei*, and *sem*), and biofilm-associated genes (*capA*, *fnbAB*, *eap*, and *fib*) [4]. In addition, genes involved in catecholamine-associated iron acquisition—such as *sstD*, *isdCEFG*, *sirABC*, *sbnABCD*, *efeO*, *feoB*, and *fur*—were identified, suggesting that enhanced iron utilization may represent a key driver of virulence. Other genes related to immune evasion during infection, including *sbi*, *efb*, and members of the *ssl* family, were also implicated. Current understanding of *S. aureus* motility remains limited. Due to the absence of flagella, motility in *S. aureus* relies on the Agr–phenol-soluble modulin (PSM) pathway, which produces surface-active molecules that enable passive spreading and dendritic expansion under highly hydrated conditions [38]. In the present study, the PSM-associated gene PFT-1 was significantly upregulated (Fig. S3). Combined with the results from clustering medium and three-layer tube assays, this suggests that the observed enhancement in clustering and chemotactic behavior may be mediated by dopamine-induced activation of the Agr–PSM pathway. Regarding protease activity, iron availability is known to play a central role in regulating both intracellular and extracellular protease expression in bacteria [39]. The observed increase in protease activity may therefore be linked to dopamine-facilitated iron acquisition, potentially through a carrier or co-carrier function, which in turn promotes the upregulation of redox-related enzymes (Fig. 2C). Finally, the growth-promoting effect observed in this study is likely multifactorial, potentially involving enhanced iron uptake, the utilization of dopamine as a nitrogen source, and its broader regulatory functions. Further investigation will be required to clarify these mechanisms.

Multiple important phenotypes of *S. aureus*, including virulence, biofilm formation, and drug resistance, are governed primarily by signal transduction systems, among which two‑component systems (e.g., SaeRS) and quorum sensing systems (e.g., Agr) play central roles [4, 40]. In this study, dopamine was found to markedly enhance a range of *S. aureus* phenotypes. These effects are likely to be closely associated with the SaeRS two‑component system, and a significant upregulation of the *agr* quorum sensing system was also observed. A limitation inherent to the knockout approach is that reduced basal expression of downstream target genes does not, by itself, distinguish between SaeRS being an essential mediator of dopamine‑triggered regulation and SaeRS acting as a general regulator of multiple virulence traits. Nonetheless, several lines of evidence point toward SaeRS as the primary route. In the WT background, dopamine specifically upregulated *saeRS* and its downstream virulence target genes, whereas in the Δ*saeS* strain the dopamine effect was diminished at some concentrations and completely abolished at others. Moreover, it is well established that many of the phenotypes examined here are under dominant, primary, or indirect control by SaeRS. It is therefore plausible that dopamine enhances *S. aureus* virulence mainly through activation of the SaeRS system. The present data, however, do not allow a definitive conclusion as to whether, and to what extent, the Agr system is also involved. As shown in Fig. S4 and Table S2, normal expression of *hld* suggests that the knockout strategy has little impact on Agr and is therefore unlikely to confound the other observations. Dopamine also significantly upregulated the Agr system, raising the possibility that the activated Agr system exerts weak or cross‑regulation on some downstream targets of SaeRS, while contributing to overlapping phenotypes—such as motility and biofilm formation—through independent pathways [5]. The specific contribution of Agr during the bacterial response to dopamine remains to be resolved, which could be addressed through genetic approaches including knockout, overexpression, and gene silencing to define the precise regulatory levels involved.

Previously, we constructed promoter–GFP reporter strains for *coa*、*hlg、fnb*、and *saeP1*, and performed fluorescence assays with WT and the Δ*saeS* mutant in the presence and absence of dopamine. However, due to signal instability and poor reproducibility, reliable data could not be obtained, and we ultimately abandoned this validation strategy [41]. Additionally, because *S. aureus* ATCC 25,923 is notoriously difficult to manipulate genetically, the knockout of *saeS* alone took nearly four months, and multiple attempts to construct a functional complemented strain were unsuccessful, which precluded more in-depth mechanistic validation. In future studies, we plan to switch to more genetically tractable strains such as Newman and USA300 to generate knockout and complemented strains [42]. We will quantitatively assess global phosphorylation levels using phosphoproteomics, and combine this with in vitro phosphorylation assays, luciferase reporter experiments, and integrative multi-omics analyses to elucidate the regulatory mechanism by which dopamine acts on the SaeRS pathway [43]. Furthermore, building on our team’s published work on *E. coli* [8], we will carry out phosphorylation and promoter–reporter validation in a Gram-negative model strain in parallel, with the aim of constructing a more complete picture of how interkingdom neurotransmitters regulate bacterial physiology. For these reasons, in the present manuscript we only present SaeRS and AgrABC as potential pathways through which dopamine modulates *S. aureus* phenotypes. We have used tentative language throughout and have drawn no definitive mechanistic conclusions; the current experimental system also cannot distinguish their individual contributions, and the mechanistic evidence remains incomplete.

The proteomic analysis differed notably from the transcriptomic approach in experimental design. Dopamine was added at the initial stage of culture in the proteomic experiments, whereas in the transcriptomic analysis it was introduced during the mid-to-late growth phase and assessed after 30 min. Despite this difference, several findings were consistent across both datasets, particularly those related to virulence, biofilm formation, and iron acquisition, including proteins such as IsdA, SbnG, EfeN, FnbAB, ClfAB, and Spa. It should be noted that protein extraction was performed using bacterial pellets, which likely limited the detection of extracellularly secreted proteins. In addition, the production of these factors generally occurs over a longer timescale than the sampling window used in this study. As a result, key virulence determinants, including enterotoxins and hemolysins, may not have been accurately captured by the proteomic analysis [44]. Although the metabolomic workflow was aligned with the transcriptomic protocol, the relatively short processing duration (30 min) suggests that dopamine-induced changes were primarily reflected at the transcriptional level. Consequently, alterations at the metabolite level were likely limited and insufficiently resolved. Taken together, these methodological constraints prevented comprehensive integration of the multi-omics datasets in the present study.

Under physiological conditions, endogenous dopamine concentrations are typically at or below the nmol/L range [45]. However, under conditions of inflammation, stress, or clinical catecholamine administration, plasma dopamine concentrations can rise several‑fold [46, 47]. Previous in vitro studies have generally employed dopamine concentrations of 5–40 µM for bacterial treatment [28]. Based on these observations, the present study selected a concentration range of 5–100 µM to investigate its regulatory effects on *S. aureus*. Future work will further refine these conditions by quantifying dopamine levels in different tissues, under stress conditions, and following pharmacological administration. Dissolved oxygen (DO) availability represents another important factor. In vivo, effective DO concentrations typically range from ≤ 0.042 to 2.12 mg O₂/L [48], and can decline markedly under pathological conditions such as stress, ischemia, shock, and sepsis, resulting in tissue hypoxia [49]. By contrast, DO levels in bacterial shake-flask cultures are approximately 6 mg O₂/L [50]. Under such conditions, even when dopamine is supplied at relatively high concentrations, reducing substances and enzymatic activity in the culture may rapidly consume available oxygen (Fig. 1C). In addition, continuous aeration and oxidation during shaking incubation complicate the relationship between the nominally added concentration (µM range) and the actual bioactive concentration. Taken together, these factors make it difficult to precisely determine the effective dopamine concentration responsible for the observed biological effects, representing an important limitation of the present study.

With respect to local concentrations in vivo, the basal striatal dopamine level has been reported as 12.0–18.0 µM in C57BL/6 mice and 15.0–22.0 µM in SD rats [51]. In dense‑core secretory vesicles of chromaffin cells and PC12 cells, dopamine is stored at concentrations around 0.1 M, whereas direct measurements in small synaptic vesicles of mouse striatal dopaminergic neurons have revealed concentrations as high as approximately 0.84 M [52]. Infection triggers the recruitment of large numbers of macrophages and neutrophils [53]; upon phagocytosis or activation by pathogen‑derived molecules, these cells can rapidly release their stored catecholamines, including dopamine [54]. At the moment of exocytosis, dopamine concentrations in the local microenvironment—such as the phagocytic synapse—can surge to tens of micromolar or even higher [55, 56]. Furthermore, estimates based on norepinephrine release from vascular sympathetic nerves indicate that the concentration within the synaptic cleft at the instant of vesicle fusion can reach 0.5–1 mM, and that over short distances the peak concentration in the interstitial fluid can still attain tens to hundreds of micromolar [57]; by analogy, a similar scenario is likely to apply to dopamine. Collectively, under physiological conditions, particularly during inflammation and stress, cells can produce substantially more catecholamines, including dopamine, than under basal conditions. Upon release, dopamine accumulates transiently in intercellular spaces and micro‑domains of infection foci, diffuses only over short distances, and very little enters the systemic circulation.

We therefore speculate that within microenvironments and micro‑tissues—such as subcutaneous sites, localized soft‑tissue infections, visceral abscesses, and the intestinal mucosa—bacteria are likely exposed to locally elevated dopamine concentrations that cannot be extrapolated from circulating levels measured under normal conditions. Under these specialized pathological structures, the local dopamine concentration may be substantially higher and may act over a longer timescale [58]. Admittedly, studies that have directly measured dopamine levels in such microscopic inflammatory foci remain scarce. Meanwhile, when the host develops severe infection, such as septic shock, high-dose catecholamine agents like dopamine may be administered to raise blood pressure, enhance myocardial contractility, and reverse organ hypoperfusion [20]. This intervention undoubtedly further elevates dopamine concentrations in certain cells and tissues, thereby potentially exacerbating bacterial infection [59].

As shown in Fig. S12, increasing dopamine concentrations in clustering medium resulted in progressively smaller colonies after dilution to single colonies, compared with the control group. This pattern was consistent in both the wild-type and knockout strains. A plausible explanation is that dopamine chelates iron ions within the clustering medium. Given the minimal fluidity of this medium, such chelation likely leads to localized iron depletion around the colonies, thereby restricting growth. In contrast, under shake-flask conditions, continuous liquid movement prevents localized iron limitation and instead supports bacterial growth (Fig. 5). Ben et al. demonstrated, using HPLC–MS and isotope ratio mass spectrometry (IRMS), that dopamine can function as an iron carrier and enter *E. coli* [8]. Together with previous findings that the SstD protein in *S. aureus* mediates the uptake of catecholamine–iron complexes, these results provide a relevant framework for interpretation. In the present study, dopamine uptake by bacterial cells was observed, and this process was enhanced by iron (Fig. 1). Coupled with the upregulation of iron transport and metabolism-related genes, these findings suggest that dopamine may also act as an iron carrier in S. aureus. To further substantiate this hypothesis, isotope-labeled experiments (e.g., using Fe-55) will be required to determine whether dopamine–iron complexes can be internalized and function in iron transport within *S. aureus* [22].

Freestone et al. reported that dopamine and norepinephrine enhance the mobilization of host-bound iron, thereby promoting bacterial recovery and reversing the inhibitory and bactericidal effects of rifampin and minocycline on *Staphylococcus* [60]. Similarly, Niu et al. demonstrated that in the presence of norepinephrine NE and Epi, the MIC values of most *E. coli* isolates for six antibiotics increased significantly [61]. Consistent with these observations, the present study found that dopamine generally elevated MIC values across multiple *S. aureus* strains and antibiotics.

Notably, this dopamine-induced increase in MIC was abolished in the *saeS* knockout strain, representing a novel finding. This observation suggests that the effect is not primarily attributable to enhanced iron acquisition counteracting antibiotic activity, but rather to regulatory mechanisms mediated by the SaeRS system. Supporting this view, Karavolos et al. showed that Salmonella can sense epinephrine and modulate antimicrobial peptide resistance via the SaeRS two-component system [62]. In addition, resistance-related genes such as *fosB*, which is regulated by the Sigma W factor and inactivates fosfomycin by cleaving its epoxy ring [63], were upregulated following dopamine treatment. In *S. aureus* ATCC 25,923, *fosB* expression increased significantly in response to dopamine but this effect was lost upon *saeS* deletion (Fig. S4), further indicating that SaeRS likely functions as a key upstream regulatory pathway. Pharmacological evidence provides additional support. Previous studies have shown that the growth-promoting effects of dopamine and norepinephrine can be blocked by phentolamine and chlorpromazine [60]. In the present study, both antagonists inhibited dopamine-induced growth promotion (Fig. S11) and attenuated the increase in MIC (Fig. 9). However, following *saeS* deletion, these antagonists exhibited minimal inhibitory effects on dopamine responses. These findings suggest that dopamine exerts its effects through multiple pathways. Its roles in promoting iron uptake and serving as a potential nitrogen source may occur independently of the signaling pathways that regulate growth and antibiotic resistance. In contrast, modulation of MIC and virulence-related phenotypes appears to depend more strongly on signal transduction mechanisms, with the SaeRS system acting as a primary regulatory component. It is therefore plausible that dopamine is sensed through receptor-mediated pathways, which can be competitively inhibited by antagonists, thereby blocking downstream signaling.

When considering the effects of *saeS* deletion alone, significant alterations were observed in the expression of multiple genes, including those associated with virulence (qPCR). However, these transcriptional changes were not consistently reflected at the phenotypic level, indicating that regulation by the SaeRS system is complex and involves additional downstream interactions. Despite this partial inconsistency, integrated analysis suggests that *saeS* is essential for the perception of dopamine or for mediating the corresponding signaling response. Dopamine may function as an exogenous signaling molecule in *S. aureus*, with its sensing and signal transduction primarily governed by the SaeRS two-component system, which in turn regulates downstream virulence-associated genes. Enterotoxin genes such as *seu* may also be influenced through this regulatory pathway. These findings are consistent with previous reports by Xiang et al., which demonstrated that dopamine can act as an exogenous signaling molecule or regulatory factor in quorum sensing, thereby promoting biofilm formation and virulence factor production in *Pseudomonas aeruginosa* PAO1 [28]. In *S. aureus*, the AgrABC system represents the central quorum-sensing network, controlling the expression of adhesion factors and global virulence programs in response to autoinducer peptide accumulation and bacterial density [64]. Given the marked upregulation of *agr* observed in this study (Fig. S4), it is likely that the Agr system also contributes to dopamine-mediated regulation. Future studies will focus on elucidating the underlying signaling mechanisms. Approaches may include molecular docking to identify potential dopamine-binding effector proteins, targeted disruption of additional components within the SaeRS system to validate its regulatory role, and genetic manipulation of the Agr system to further define its contribution to dopamine-dependent responses.

Considering the effects of dopamine treatment on the phenotype of the *saeS* knockout strain, the absence of *saeS* led to the loss of most dopamine-induced enhancements observed in the WT strain. This finding suggests that signaling-mediated regulation plays a more dominant role than iron-carrier activity in this context. However, within the host environment, iron availability is tightly restricted. Host proteins such as transferrin and lactoferrin sequester Fe³⁺, forming an effective iron barrier [11]. In contrast, TSB medium used in this study does not represent an iron-limited environment. Previous studies have frequently employed SAPI medium, a nutrient-limited and iron-deficient system supplemented with animal serum, to better simulate in vivo conditions [65]. Therefore, it remains unclear whether the role of dopamine as an iron carrier is attenuated under standard culture conditions, or whether mechanisms identified in TSB medium may be partially obscured or altered in SAPI-like environments. Microbial endocrinology is an emerging interdisciplinary field that remains in its early stages of development [66]. Current research generally encompasses three main directions: in vitro studies that simulate host-like microenvironments to investigate the direct regulatory effects of hormones on pathogens [9]; in vivo approaches that utilize host stress models to examine how endogenous hormones influence microbial community structure and function [67]; and, more recently, studies exploring bidirectional interactions, particularly how microbial communities in turn modulate host physiological states [68].

SaeS serves not only as a central component required for maintaining the function of the SaeRS two-component system but also as a key regulator of *S. aureus* pathogenicity, making it a promising target for anti-virulence therapeutic strategies [4, 37]. Similar approaches may also be extended to other bacterial two-component systems. In the present study, deletion of the *saeS* gene affected multiple phenotypic traits, including virulence, and dopamine supplementation failed to markedly enhance virulence in the knockout strain. These findings further support the essential role of SaeS in mediating dopamine-dependent pathogenic responses. Given the growing challenge of bacterial resistance, as well as the need to mitigate infection risks under host stress conditions or during elevated systemic catecholamine levels, targeting two-component regulatory systems may represent an effective strategy to reduce pathogen virulence and infection potential. In addition, the modulation of antibiotic resistance by exogenous dopamine observed in this study has important clinical and scientific implications. Notably, resistance to streptomycin sulfate and several other antibiotics was reduced in certain *S. aureus* strains under specific conditions. This raises the possibility that combining dopamine with selected antimicrobial agents may offer a novel approach for managing particular forms of antibiotic resistance [69].

Multiple studies have shown that exogenous epinephrine and norepinephrine induce oxidative stress and upregulate associated genes in bacteria, thereby inhibiting bacterial proliferation [12]. In contrast, the present study observed effects that are generally supportive of bacterial proliferation and other phenotypes. Nevertheless, under in vitro conditions—particularly at high dissolved oxygen levels and high dopamine concentrations—there is a reasonable concern that reactive oxygen species (ROS) may be generated and cause cellular damage [70]. We therefore measured ROS levels in *S. aureus* using the same treatment protocol as for our transcriptomic analysis, with H₂O₂ serving as a positive control. Concurrently, we re‑examined the expression of genes associated with oxidative stress, including *saeRS*, *agrABC*, and *ahpCF* (encoding alkyl hydroperoxide reductase). Transcriptomic analysis did not reveal any obvious enrichment of oxidative stress‑related pathways. As shown in Fig. S13, dopamine treatment for 30 min did not cause a significant increase in ROS levels compared with the control. Notably, similar results were obtained not only for *S. aureus* ATCC 25,923, but also for *E. coli* ATCC 11,303, *E. coli* ATCC 35,150, and *Serratia marcescens* ATCC 13,880. Fig. S14 shows that, across the tested concentrations, dopamine did not induce appreciable changes in the transcript levels of DNA damage repair genes; only *ahpCF* was modestly upregulated (1.5‑fold). In contrast, H₂O₂ strongly activated the ROS‑scavenging gene *ahpCF* and upregulated all examined DNA repair genes in a dose‑dependent manner, indicative of severe oxidative damage. Thus, dopamine did not significantly activate oxidative stress or DNA damage responses, standing in clear contrast to the broad‑spectrum oxidative stress pattern induced by H₂O₂. Collectively, these findings indicate that ROS is not a key contributing factor under our experimental conditions, although we acknowledge that measurements at additional time points and a more comprehensive evaluation of the role of ROS are warranted in future studies.

## Conclusion

This study demonstrates that dopamine can be internalized by *S. aureus*, where it markedly enhances bacterial growth, virulence, biofilm formation, motility, host adhesion, and multidrug resistance. Multi-omics analyses indicate that dopamine primarily activates the SaeRS two-component system, leading to the upregulation of key energy metabolism pathways, including glucose metabolism and oxidative phosphorylation. In parallel, the expression of virulence-associated factors and nitrogen metabolism-related genes is significantly increased, accompanied by moderate activation of membrane lipid biosynthesis and cell envelope remodeling processes. Deletion of *saeS* markedly attenuated the pro‑virulence effect of dopamine, whereas dopamine simultaneously activated the Agr pathway, suggesting that this regulatory process is likely driven primarily by SaeRS with a cooperative contribution from Agr.

## Supplementary Information

Supplementary Material 1.

## Data Availability

Relevant data are available within this manuscript. Moreover, all raw omics data generated in this study have been publicly deposited in the National Genomics Data Center (NGDC, China National Center for Bioinformation). The transcriptome sequencing data are available in the Genome Sequence Archive (GSA) under accession number CRA042114 (available at https://ngdc.cncb.ac.cn/gsa/browse/CRA042114). The mass spectrometry proteomics data have been deposited to the ProteomeXchange Consortium (https://proteomecentral.proteomexchange.org) via the iProX partner repository with the dataset identifier PXD078195. The mass spectrometry-based metabolomic data are uploaded to OMIX under accession number OMIX016583 (available at https://ngdc.cncb.ac.cn/omix/release/OMIX016583). All datasets are affiliated with the BioProject PRJCA063171 and can be freely accessed and downloaded without restrictions.

## References

[1] Kaasch AJ, Michels G. Staphylococcus aureus Bloodstream Infection: When Is Transthoracic Echocardiography Sufficient? JACC Cardiovasc Imaging. 2015;8(8):932–3. 10.1016/j.jcmg.2015.02.025. 26271089

[2] Cheung GYC, Bae JS, Otto M. Pathogenicity and virulence of Staphylococcus aureus. Virulence. 2021;12(1):547–69. 10.1080/21505594.2021.1878688. PMC7872022 33522395

[3] Hao L, Zhou J, yang H, He C, Shu W, Song H, et al. Anti-virulence potential of iclaprim, a novel folic acid synthesis inhibitor, against Staphylococcus aureus. Appl Microbiol Biotechnol. 2024;108(1). 10.1007/s00253-024-13268-2. PMC11300511 39102054

[4] Liu Q, Yeo WS, Bae T. The SaeRS Two-Component System of Staphylococcus aureus. Genes. 2016;7(10). 10.3390/genes7100081.

[5] Stampfl M, DeBlieux P. A Clinical Review of Vasopressors in Emergency Medicine. J Emerg Med. 2024;67(1):e31–41. 10.1016/j.jemermed.2024.03.004. 38789351

[6] Kislitsina ON, Rich JD, Wilcox JE, Pham DT, Churyla A, Vorovich EB, et al. Shock – Classification and Pathophysiological Principles of Therapeutics. Curr Cardiol Rev. 2019;15(2):102–13. 10.2174/1573403x15666181212125024. PMC6520577 30543176

[7] Pfister D, Strebel SP, Steiner LA. Effects of glyceryl trinitrate on cerebrovascular autoregulation. Br J Anaesth. 2008;101(4):574. 10.1093/bja/aen254. author reply – 5. 18782889

[8] Xu B, Chen X, Chai J, Wei Y. Dopamine Is a Key Regulatory Molecule for Escherichia coli and May Serve as a Xenosiderophore. Microorganisms. 2026;14(2). 10.3390/microorganisms14020327. PMC12943675 41753614

[9] Boujnane M, Boukerb AM, Connil N. Bacterial gene expression in response to catecholamine stress hormones. Curr Opin Endocr Metabolic Res. 2024;36. 10.1016/j.coemr.2024.100543.

[10] Melin V, Henríquez A, Freer J, Contreras D. Reactivity of catecholamine-driven Fenton reaction and its relationships with iron(III) speciation. Redox Rep. 2014;20(2):89–96. 10.1179/1351000214y.0000000119. PMC6837720 25496478

[11] Sandrini SM, Shergill R, Woodward J, Muralikuttan R, Haigh RD, Lyte M, et al. Elucidation of the Mechanism by Which Catecholamine Stress Hormones Liberate Iron from the Innate Immune Defense Proteins Transferrin and Lactoferrin. J Bacteriol. 2010;192(2):587–94. 10.1128/jb.01028-09. PMC2805316 19820086

[12] Luqman A. The orchestra of human bacteriome by hormones. Microb Pathog. 2023;180. 10.1016/j.micpath.2023.106125. 37119938

[13] Perraud Q, Kuhn L, Fritsch S, Graulier G, Gasser V, Normant V, et al. Opportunistic use of catecholamine neurotransmitters as siderophores to access iron by Pseudomonas aeruginosa. Environ Microbiol. 2021;24(2):878–93. 10.1111/1462-2920.15372. 33350053

[14] Zhang Y, Edmonds KA, Raines DJ, Murphy BA, Wu H, Guo C, et al. The Pneumococcal Iron Uptake Protein A (PiuA) Specifically Recognizes Tetradentate FeIIIbis- and Mono-Catechol Complexes. J Mol Biol. 2020;432(19):5390–410. 10.1016/j.jmb.2020.08.005. PMC7502554 32795535

[15] Beasley FC, Marolda CL, Cheung J, Buac S, Heinrichs DE, Payne SM. Staphylococcus aureus Transporters Hts, Sir, and Sst Capture Iron Liberated from Human Transferrin by, Staphyloferrin A, Staphyloferrin B. and Catecholamine Stress Hormones, Respectively, and Contribute to Virulence. Infection and Immunity. 2011;79(6):2345-55; 10.1128/iai.00117-11 PMC3125851 21402762

[16] Freestone PPE, Sandrini SM, Haigh RD, Lyte M. Microbial endocrinology: how stress influences susceptibility to infection. Trends Microbiol. 2008;16(2):55–64. 10.1016/j.tim.2007.11.005. 18191570

[17] Sun H, Huang D, Pang Y, Chen J, Kang C, Zhao M, et al. Key roles of two-component systems in intestinal signal sensing and virulence regulation in enterohemorrhagic Escherichia coli. FEMS Microbiol Rev. 2024;48(6). 10.1093/femsre/fuae028. PMC11644481 39537200

[18] Dicks LMT. Gut Bacteria and Neurotransmitters. Microorganisms. 2022;10(9):1838. 10.3390/microorganisms10091838 PMC9504309 36144440

[19] Verbrugghe E, Boyen F, Gaastra W, Bekhuis L, Leyman B, Van Parys A, et al. The complex interplay between stress and bacterial infections in animals. Vet Microbiol. 2012;155(2–4):115–27. 10.1016/j.vetmic.2011.09.012. 21963418

[20] Li X, Cheng L, Liu X, Wang X, Li R, Fan S, et al. Dopamine promotes Klebsiella quasivariicola proliferation and inflammatory response in the presence of macrophages. Front Cell Infect Microbiol. 2024;14. 10.3389/fcimb.2024.1322113. PMC10995343 38585654

[21] Treangen TJ, Maybank RA, Enke S, Friss MB, Diviak LF, Karaolis DKR, et al. Complete Genome Sequence of the Quality Control Strain Staphylococcus aureus subsp. Genome Announcements. 2014;2(6). 10.1128/genomeA.01110-14. PMC4223452 25377701

[22] Beal J, Farny NG, Haddock-Angelli T, Selvarajah V, Baldwin GS, Buckley-Taylor R, et al. Robust estimation of bacterial cell count from optical density. Commun Biology. 2020;3(1):512. 10.1038/s42003-020-01127-5. PMC7499192 32943734

[23] Pollack-Milgate S, Saitia S, Tang JX. Rapid growth rate of Enterobacter sp. SM3 determined using several methods. BMC Microbiol. 2024;24(1):403. 10.1186/s12866-024-03547-3. PMC11465882 39390418

[24] Ponnuswamy V. A simple method for the detection of protease activity on agar plates using bromocresolgreen dye. J Biochem Technol. 2013.

[25] Grossman AB, Burgin DJ, Rice KC. Quantification of Staphylococcus aureus Biofilm Formation by Crystal Violet and Confocal Microscopy. Methods Mol Biol. 2021;2341:69–78. 10.1007/978-1-0716-1550-8_9. 34264462

[26] Cheung GYC, Duong AC, Otto M. Direct and synergistic hemolysis caused by Staphylococcus phenol-soluble modulins: implications for diagnosis and pathogenesis. Microbes Infect. 2012;14(4):380–6. 10.1016/j.micinf.2011.11.013. PMC3299937 22178792

[27] Edwin C. Quantitative determination of staphylococcal enterotoxin A by an enzyme-linked immunosorbent assay using a combination of polyclonal and monoclonal antibodies and biotin-streptavidin interaction. J Clin Microbiol. 1989;27(7):1496–501. 10.1128/jcm.27.7.1496-1501.1989. PMC267602 2768439

[28] Xiang S-L, Xu K-Z, Yin L-J, Rao Y, Wang B, Jia A-Q. Dopamine, an exogenous quorum sensing signaling molecule or a modulating factor in Pseudomonas aeruginosa? Biofilm. 2024;8. 10.1016/j.bioflm.2024.100208. PMC11260039 39036334

[29] Zheng Y, Li Y, Past, Present. Future of Tools for Dopamine Detection. Neuroscience. 2023;525:13–25. 10.1016/j.neuroscience.2023.06.025. 37419404

[30] Monk IR, Stinear TP. From cloning to mutant in 5 days: rapid allelic exchange in Staphylococcus aureus. Access Microbiol. 2021;3(2):000193. 10.1099/acmi.0.000193. PMC8209637 34151146

[31] Audrey N, Schuetz nbsp, Ferrell A, Janet nbsp, Hindler A. Overview of changes in the Clinical and Laboratory Standards Institute Performance Standards for Antimicrobial Susceptibility Testing: M100 32nd and 33rd editions. J Clin Microbiol. 2025;e0162323. 10.1128/jcm.01623-23. PMC12421849 40772786

[32] Letourneau J, Levesque C, Berthiaume F, Jacques M, Mourez M. In vitro assay of bacterial adhesion onto mammalian epithelial cells. J Vis Exp. 2011;(51):2783. 10.3791/2783. PMC3197129 21633326

[33] Dong Y, Liu J, Pang M, Du H, Wang N, Awan F, et al. Catecholamine-Stimulated Growth of Aeromonas hydrophila Requires the TonB2 Energy Transduction System but Is Independent of the Amonabactin Siderophore. Front Cell Infect Microbiol. 2016;6. 10.3389/fcimb.2016.00183. PMC5149522 28018865

[34] Nguyen JNT, Harbison AM. Scanning Electron Microscopy Sample Preparation and Imaging. In: Espina V, editor. Molecular Profiling: Methods and Protocols. New York, NY: Springer New York; 2017. pp. 71–84. 10.1007/978-1-4939-6990-6_5 28501994

[35] Feng J, Zheng Y, Ma W, Weng D, Peng D, Xu Y, et al. A synthetic antibiotic class with a deeply-optimized design for overcoming bacterial resistance. Nat Commun. 2024;15(1):6040. . 10.1038/s41467-024-50453-3 PMC11255307 39019927

[36] Curis E, Nepost C, Grillault Laroche D, Courtin C, Laplanche J-L, Etain B, et al. Selecting reference genes in RT-qPCR based on equivalence tests: a network based approach. Sci Rep. 2019;9(1):16231. 10.1038/s41598-019-52217-2. PMC6838083 31700128

[37] Yeaman MR, Liu Q, Cho H, Yeo W-S, Bae T. The Extracytoplasmic Linker Peptide of the Sensor Protein SaeS Tunes the Kinase Activity Required for Staphylococcal Virulence in Response to Host Signals. PLoS Pathog. 2015;11(4). 10.1371/journal.ppat.1004799. PMC4388633 25849574

[38] Jacob KM, Hernández-Villamizar S, Hammer ND, Reguera G. Mucin-induced surface dispersal of < i>Staphylococcus aureus and *Staphylococcus epidermidis* via quorum-sensing dependent and independent mechanisms. mBio. 2024;15(8). 10.1128/mbio.01562-24. PMC11323471 38953351

[39] Johnson M, Sengupta M, Purves J, Tarrant E, Williams PH, Cockayne A, et al. Fur is required for the activation of virulence gene expression through the induction of the sae regulatory system in Staphylococcus aureus. Int J Med Microbiol. 2011;301(1):44–52. 10.1016/j.ijmm.2010.05.003. PMC2994983 20705504

[40] Tan L, Li SR, Jiang B, Hu XM, Li S. Therapeutic Targeting of the Staphylococcus aureus Accessory Gene Regulator (agr) System. Front Microbiol. 2018;9. 10.3389/fmicb.2018.00055. PMC5789755 29422887

[41] Anderson EE, Dyzenhaus S, Ilmain JK, Sullivan MJ, van Bakel H, Torres VJ. SarS Is a Repressor of Staphylococcus aureus Bicomponent Pore-Forming Leukocidins. Infect Immun. 2023;91(4):e0053222. 10.1128/iai.00532-22. PMC10112191 36939325

[42] Fey Paul D, Endres Jennifer L, Yajjala Vijaya K, Widhelm Todd J, Boissy Robert J, Bose Jeffrey L, et al. A Genetic Resource for Rapid and Comprehensive Phenotype Screening of Nonessential Staphylococcus aureus Genes. mBio. 2013;4(1). 10.1128/mbio.00537. PMC3573662 23404398

[43] Sun F, Li C, Jeong D, Sohn C, He C, Bae T. In theStaphylococcus aureusTwo-Component Systemsae, the Response Regulator SaeR Binds to a Direct Repeat Sequence and DNA Binding Requires Phosphorylation by the Sensor Kinase SaeS. J Bacteriol. 2010;192(8):2111–27. 10.1128/jb.01524-09. PMC2849438 20172998

[44] Busche T, Hillion M, Van Loi V, Berg D, Walther B, Semmler T, et al. Comparative Secretome Analyses of Human and Zoonotic Staphylococcus aureus Isolates CC8, CC22, and CC398. Mol Cell Proteom. 2018;17(12):2412–33. 10.1074/mcp.RA118.001036. PMC6283302 30201737

[45] van der Hoorn FA, Boomsma F, Veld Min’t AJ, Schalekamp MA. Determination of catecholamines in human plasma by high-performance liquid chromatography: comparison between a new method with fluorescence detection and an established method with electrochemical detection. J Chromatogr. 1989;487(1):17–28. 10.1016/s0378-4347(00)83003-0. 2715261

[46] Petrulli JR, Kalish B, Nabulsi NB, Huang Y, Hannestad J, Morris ED. Systemic inflammation enhances stimulant-induced striatal dopamine elevation. Transl Psychiatry. 2017;7(3):e1076. 10.1038/tp.2017.18. PMC5404612 28350401

[47] Shimojo G, Joseph B, Shah R, Consolim-Colombo FM, De Angelis K, Ulloa L. Exercise activates vagal induction of dopamine and attenuates systemic inflammation. Brain Behav Immun. 2019;75:181–91. 10.1016/j.bbi.2018.10.005. PMC6334665 30394312

[48] Zheng L, Kelly CJ, Colgan SP. Physiologic hypoxia and oxygen homeostasis in the healthy intestine. A Review in the Theme: Cellular Responses to Hypoxia. Am J Physiology-Cell Physiol. 2015;309(6):C350–60. 10.1152/ajpcell.00191.2015. PMC4572369 26179603

[49] Ripa C, Munshi L, Kuebler WM, Magliocca A, Taccone FS, Ware LB, et al. Oxygen targets in critically ill patients: from pathophysiology to population enrichment strategies. Med Gas Res. 2025;15(3):409–19. 10.4103/mgr.MEDGASRES-D-24-00120. PMC12054680 40251021

[50] Vendruscolo F, Rossi MJ, Schmidell W, Ninow JL. Determination of Oxygen Solubility in Liquid Media. ISRN Chem Eng. 2012;2012:1–5. 10.5402/2012/601458.

[51] Zeng X-S, Geng W-S, Jia J-J. Neurotoxin-Induced Animal Models of Parkinson Disease: Pathogenic Mechanism and Assessment. ASN Neuro. 2018;10(1). 10.1177/1759091418777438. PMC5977437 29809058

[52] Omiatek DM, Bressler AJ, Cans A-S, Andrews AM, Heien ML, Ewing AG. The real catecholamine content of secretory vesicles in the CNS revealed by electrochemical cytometry. Sci Rep. 2013;3(1). 10.1038/srep01447. PMC3596796 23486177

[53] Xue J, Lin Y, Oo D, Zhang J, Zardynezhad A, de Jesus FN et al. Lymph-derived chemokines direct early neutrophil infiltration in the lymph nodes upon < i>Staphylococcus aureus skin infection. Proceedings of the National Academy of Sciences. 2022;119(32):e2111726119; doi: 10.1073/pnas.2111726119 PMC9371737 35914162

[54] Kopin IJ, Zukowska-Grojec Z, Bayorh MA, Goldstein DS. Estimation of intrasynaptic norepinephrine concentrations at vascular neuroeffector junctions in vivo. Naunyn Schmiedebergs Arch Pharmacol. 1984;325(4):298–305. 10.1007/BF00504372. 6728040

[55] Cosentino M, Marino F, Maestroni GJM. Sympathoadrenergic modulation of hematopoiesis: a review of available evidence and of therapeutic perspectives. Front Cell Neurosci. 2015;9. 10.3389/fncel.2015.00302. PMC4525045 26300737

[56] Ch Beck G, Brinkkoetter P, Hanusch C, Schulte J, van Ackern K, van der Woude FJ, et al. Crit Care. 2004;8(6). 10.1186/cc2879. PMC1065039 15566620

[57] Goldstein DS, Zimlichman R, Stull R, Keiser HR, Kopin IJ. Estimation of intrasynaptic norepinephrine concentrations in humans. Hypertension. 1986;8(6):471–5. 10.1161/01.HYP.8.6.471. 3710555

[58] Oleskin AV, Sorokina EV, Shilovsky GA. Interaction of Catecholamines with Microorganisms, Neurons, and Immune Cells. Biology Bull Reviews. 2021;11(4):358–67. 10.1134/S2079086421040058.

[59] Hartemink KJ, Groeneveld AB. Vasopressors and inotropes in the treatment of human septic shock: effect on innate immunity? Inflammation. 2012;35(1):206–13. 10.1007/s10753-011-9306-8. PMC3282003 21347606

[60] Freestone Primrose PE, Haigh Richard D, Lyte M. Catecholamine Inotrope Resuscitation of Antibiotic-Damaged Staphylococci and Its Blockade by Specific Receptor Antagonists. J Infect Dis. 2008;197(7):1044–52. 10.1086/529202. 18419472

[61] Niu L, Gao M, Wen S, Wang F, Shangguan H, Guo Z, et al. Effects of Catecholamine Stress Hormones Norepinephrine and Epinephrine on Growth, Antimicrobial Susceptibility, Biofilm Formation, and Gene Expressions of Enterotoxigenic Escherichia coli. Int J Mol Sci. 2023;24(21). 10.3390/ijms242115646. PMC10649963 37958634

[62] Karavolos MH, Spencer H, Bulmer DM, Thompson A, Winzer K, Williams P, et al. Adrenaline modulates the global transcriptional profile of Salmonella revealing a role in the antimicrobial peptide and oxidative stress resistance responses. BMC Genomics. 2008;9(1):458. 10.1186/1471-2164-9-458. PMC2576261 18837991

[63] Aiezza N, Antonelli A, Coppi M, Di Pilato V, Giani T, Rossolini GM. Up-regulation of resident chromosomal fosB gene expression: a novel mechanism of acquired fosfomycin resistance in MRSA. J Antimicrob Chemother. 2023;78(7):1599–605. 10.1093/jac/dkad126. 37161536

[64] Chen L, Huang M, Chen Y, Lin Y, Cong Y. Research Progress on Antivirulence Agents Targeting the Accessory Gene Regulator (Agr) System of Staphylococcus Aureus. Infect Drug Resist. 2026;19:1–13. 10.2147/idr.S594449. PMC13032729 41908596

[65] Sarkodie EK, Zhou S, Baidoo SA, Chu W. Influences of stress hormones on microbial infections. Microb Pathog. 2019;131:270–6. 10.1016/j.micpath.2019.04.013. 30981718

[66] Feuilloley MGJ, Lesouhaitier O. Endocrine microbiology: A transdisciplinary approach of the human physiology. Curr Opin Endocr Metabolic Res. 2025;38:100570. 10.1016/j.coemr.2025.100570.

[67] Langgartner D, Peterlik D, Foertsch S, Füchsl AM, Brokmann P, Flor PJ, et al. Individual differences in stress vulnerability: The role of gut pathobionts in stress-induced colitis. Brain Behav Immun. 2017;64:23–32. 10.1016/j.bbi.2016.12.019. 28012830

[68] Xiang G, Wang Y, Ni K, Luo H, Liu Q, Song Y, et al. Nasal Staphylococcus aureus carriage promotes depressive behaviour in mice via sex hormone degradation. Nat Microbiol. 2025;10(10):2425–40. 10.1038/s41564-025-02120-6. PMC12488488 40983700

[69] Bognár B, Spohn R, Lázár V. Drug combinations targeting antibiotic resistance. npj Antimicrobials Resist. 2024;2(1):29. 10.1038/s44259-024-00047-2. PMC11721080 39843924

[70] Umek N, Geršak B, Vintar N, Šoštarič M, Mavri J. Dopamine Autoxidation Is Controlled by Acidic pH. Front Mol Neurosci. 2018;11–2018. 10.3389/fnmol.2018.00467. PMC6305604 30618616

