# Supplementary material for: The SaeRS two-component system likely mediates the major dopamine-dependent regulation of virulence in Staphylococcus aureus ATCC 25,923
Source: BMC Microbiol. 2026 Aug 18;26:1005. doi: 10.1186/s12866-026-05427-4 (PMC13760889; doi:10.1186/s12866-026-05427-4)
Supplement: Supplementary file 1 [file 12866_2026_5427_MOESM1_ESM.zip › Supplementary material/Animal Ethics and Welfare/Approval of Animal Ethical and Welfare.PDF]

动物实验伦理审查报告

Approval of Animal Ethical and Welfare

四川里来思诺生物科技有限公司实验动物伦理委员会对拟开展项目的相关伦理学问题进行了审查。

The Ethics Committee of Sichuan Lilaisinuo Biological Technology Co., LTD reviewed the ethical issues related to the proposed research project.

伦理备案号	LLSN-2024265	Ethical No.	LLSN-2024265
项目名称<br>Protocol Title	测试多巴胺对于金葡菌侵袭小鼠的作用<br>To investigate the effects of dopamine on the invasion of *Staphylococcus aureus* in mice		
申请人单位<br>Department	昆明理工大学生命科学与技术学院 B103 课题组<br>Faculty of Life Science and Technology, Kunming University of Science and Technology		

项目负责人<br>Principle Investigator (PI)	魏云林<br>Yunlin Wei	职称<br>Professional	教授<br>Professor	联系电话<br>Tel	13888644138
联系人<br>Contacts	徐奔	联系电话<br>Tel	13093575059	邮箱<br>E-mail	xuben6616@163.com

拟研究时间： 2025 年 1 月 10 日至 2025 年 1 月 18 日

Period of Protocol: From：January 10, 2025 To：January 18, 2025

递交审查资料：☑研究方案 ☑其他资料

（其他资料包括：试验用品安全性资料、生产企业资质证明、试验用品提供者的资质证明等）

Submission of examination materials: ☑Study Protocol ☑Other materials

(Other materials include: safety data of test supplies, qualification certificate of production enterprise, qualification certificate of test supplies provider, etc.)

动物实验申请陈述<br>Statement of application for animal experiment	动物品系/品种/性别/年龄/体重	8 周雌性 ICR 小鼠	实验动物有无离室再入室要求	无
	选择使用动物数量依据	统计学要求	有无繁殖动物的要求	无
	动物设施要求	SPF 级	动物是否安乐死/安乐死方法	是/过量异氟醚吸入法
	手术间或动物实验室要求	SPF 级	动物麻醉方法	2%戊巴比妥 (35mg/kg)
	有无特殊动物饲养要求	无	动物尸体处理方法	统一交由四川里来思诺生物科技有限公司进行无害化处理

研究方案简介<br>Introduction to the research scheme	ICR小鼠适应性饲养五天后，进行灌胃处理，分别给药PBS及盐酸多巴胺。之后给予小鼠菌液灌胃处理，正常饲养后，解剖，取内脏，采用平板稀释法测内脏细菌载量，来判定多巴胺对于金葡菌侵袭的影响。<br>After 5 days of acclimatization, ICR mice were subjected to gavage administration with PBS and Dopamine hydrochloride, respectively. Subsequently, the mice were gavaged with bacterial suspension. Following normal feeding, the mice were euthanized and dissected to collect visceral organs. The plate dilution method was used to determine the visceral bacterial load, so as to evaluate the effect of dopamine on the invasion of Staphylococcus aureus.

申请人（项目负责人）承诺：

The applicant (project leader) promises:

以上所填内容（包括各附件材料）均属实，如获批准，我将严格按照提供的方案进行研究，并遵守四川里来思诺生物科技有限公司实验动物伦理委员会的相关规定。

The above information (including the attached materials) is true. If approved, I will study in strict accordance with the provided program and abide by the relevant regulations of ethics committee of Sichuan Lilaisinuo Biological Technology Co., LTD.

申请人（项目负责人）签字：魏云林　　日期：2024 年 12 月 20 日

Signature of applicant (project leader): Yunlin Wei　　Date: 2024-12-20

审查结果<br>Review result	☑同意<br>□Agreement	□不同意<br>□Disagreement	□修改<br>□Modification

伦理委员会审批意见：

Review and approval opinions of ethics committee:

经我单位伦理委员会审议，该研究的实验设计和方案充分考虑了安全性和公平性原则，该项目研究内容和过程中所涉及的实验动物，均符合国家对医学实验动物的有关要求，同意该项目的研究工作按计划进行。

After the review by the ethics committee of our unit, the experimental design and scheme of this research fully consider the principle of safety and fairness. The research content and experimental animals involved in the process of this project are in line with the relevant requirements of the state on medical experimental animals, and the research work of this project is agreed to proceed as planned.

主任委员（签章）：阳丹丹　　日期: 2024-12-20　　四川里来思诺实验动物伦理委员会（盖章）

Chairman: (signature) Dandan Yang　　Date: 2024-12-20　　The Ethics Committee of Sichuan Lilaisinuo (seal)

四川里来思诺生物科技有限公司 实验动物伦理委员会

生物科技有限公司 伦理委员会
