# Supplementary material for: The SaeRS two-component system likely mediates the major dopamine-dependent regulation of virulence in Staphylococcus aureus ATCC 25,923
Source: BMC Microbiol. 2026 Aug 18;26:1005. doi: 10.1186/s12866-026-05427-4 (PMC13760889; doi:10.1186/s12866-026-05427-4)
Supplement: Supplementary file 1 [file 12866_2026_5427_MOESM1_ESM.zip › Supplementary material/Animal Ethics and Welfare/Test Platform Qualification Certificate (Original Chinese Version)/Business License.pdf]

统一社会信用代码

91510115MA69GEMP68

营业执照

（副　本）　副本编号：1 - 1

扫描经营主体信息码，了解更多登记、备案、许可、监管信息。

名　　称　四川里来思诺生物科技有限公司

注册资本　壹佰万元整

类　　型　有限责任公司(自然人投资或控股)

成立日期　2020年12月18日

法定代表人　王蓉

住　　所　成都市温江区成都海峡两岸科技产业开发园双堰路1919号3号楼1-3层

经营范围　一般项目：技术服务、技术开发、技术咨询、技术交流、技术转让、技术推广；工程和技术研究和试验发展；生物基材料技术研发；翻译服务；会议及展览服务；自然科学研究和试验发展；医学研究和试验发展。（除依法须经批准的项目外，凭营业执照依法自主开展经营活动）许可项目：检验检测服务；医疗服务；药品互联网信息服务；医疗器械互联网信息服务。（依法须经批准的项目，经相关部门批准后方可开展经营活动，具体经营项目以相关部门批准文件或许可证件为准）

登记机关

成都市温江区市场监督管理局

2025　年 11　月 12　日

国家企业信用信息公示系统网址：http://www.gsxt.gov.cn

市场主体应当于每年1月1日至6月30日通过国家企业信用信息公示系统报送公示年度报告。

国家市场监督管理总局监制

仅供报账使用
