# Supplementary material for: The SaeRS two-component system likely mediates the major dopamine-dependent regulation of virulence in Staphylococcus aureus ATCC 25,923
Source: BMC Microbiol. 2026 Aug 18;26:1005. doi: 10.1186/s12866-026-05427-4 (PMC13760889; doi:10.1186/s12866-026-05427-4)
Supplement: Supplementary file 1 [file 12866_2026_5427_MOESM1_ESM.zip › Supplementary material/Animal Ethics and Welfare/Test Platform Qualification Certificate (Original Chinese Version)/Proficiency Testing Certificate.pdf]

检验检测机构
资质认定证书

证书编号：222303101420

名称：四川里来思诺生物科技有限公司

地址：成都市温江区成都海峡两岸科技产业开发园双堰路1919号3号楼1-3层

经审查，你机构已具备国家有关法律、行政法规规定的基本条件和能力，现予批准，可以向社会出具具有证明作用的数据和结果，特发此证。资质认定包括检验检测机构计量认证。

检验检测能力及授权签字人见证书附表。

你机构对外出具检测报告或证书的法律责任由四川里来思诺生物科技有限公司承担。

许可使用标志

CMA

222303101420

发证日期：2022年10月20日

有效期至：2028年10月19日

发证机关：四川省市场监督管理局

本证书由国家认证认可监督管理委员会监制，在中华人民共和国境内有效。

仅作为报账使用，再次复印无效
