# Supplementary material for: The SaeRS two-component system likely mediates the major dopamine-dependent regulation of virulence in Staphylococcus aureus ATCC 25,923
Source: BMC Microbiol. 2026 Aug 18;26:1005. doi: 10.1186/s12866-026-05427-4 (PMC13760889; doi:10.1186/s12866-026-05427-4)
Supplement: Supplementary file 1 [file 12866_2026_5427_MOESM1_ESM.zip › Supplementary material/Animal Ethics and Welfare/Test Platform Qualification Certificate (Original Chinese Version)/Qualification Certificate for Inspection Body.pdf]

中国认可
能力验证
PROFICIENCY TESTING
CNAS PT0031

能力验证合格实验室证书

编号：2205-01

四川里来思诺生物科技有限公司：

您实验室参加中国合格评定国家认可委员会能力验证计划提供者组织的 PT0031-2205 毒性病理学检查 能力验证项目，其中 毒性病理学诊断 检测参数，提交的测试结果为 满意 ，特发此证。

2022年12月16日

宁波海关技术中心能力验证专用章（4）

宁波海关技术中心

仅供报账使用
