# Supplementary material for: The SaeRS two-component system likely mediates the major dopamine-dependent regulation of virulence in Staphylococcus aureus ATCC 25,923
Source: BMC Microbiol. 2026 Aug 18;26:1005. doi: 10.1186/s12866-026-05427-4 (PMC13760889; doi:10.1186/s12866-026-05427-4)
Supplement: Supplementary file 1 [file 12866_2026_5427_MOESM1_ESM.zip › Supplementary material/HPLC-MS Materials and Methods.docx]

*1. High - Performance Liquid Chromatography-Mass (HPLC-MS)*

*1.1. Instruments and Reagents*

| Instruments and reagents | Producer |
| --- | --- |
| Ultra-High Performance Liquid Chromatography （HCLASS） | Waters, Milford, USA |
| Triple Quadrupole Mass Spectrometry （AB 4500） | AB Sciex, Suzhou, China |
| Chromatographic column（BEH Amide 1.7um 3.0*100mm） | Waters, Milford, USA |
| Methanol | Merck, Darmstadt, Germany |
| Ultrapure water | Merck, Darmstadt, Germany |
| Acetonitrile | Merck, Darmstadt, Germany |
| Formic acid | Merck, Darmstadt, Germany |

*1.1. Methods*

Sample Preparation.

Add 100 μL of sample to 300 μL of methanol and vortex for 60 seconds. Centrifuge at 17,000×g for 15 minutes, and collect the supernatant for subsequent use.

Sample and Standard Curve Preparation.

Dilute the internal standard (IS) with 75% methanol/water to a specific concentration. Mix the standards and samples with the IS solution at a 1:1 volume ratio for dilution. Prepare the calibration curve diluent by diluting the IS solution with 75% methanol/water at a 1:1 volume ratio, followed by serial dilution to specific concentrations. The final concentration of the IS is 250 ng/mL.

*1.2. Instrument Parameters*

Column temperature: 40 °C; injection volume: 5 μL. Positive ion mode: Mobile phase B: 0.1% formic acid in water (containing 10 mM ammonium formate).

Mobile phase D: 90% acetonitrile (containing 0.2% formic acid and 10 mM ammonium formate).

*1.3. Mobile Phase Elution Gradient*

| Time(min) | Flow-rate  (μl /min) | B% | D% |
| --- | --- | --- | --- |
| 0.00 | 0.400 | 10 | 90 |
| 10.00 | 0.400 | 10 | 90 |

*1.4. Mass Spectrometry Conditions*

Data acquisition of primary and secondary mass spectrometry was performed on an AB 4500 mass spectrometer in multiple reaction monitoring (MRM) mode with negative electrospray ionization (ESI) source. The ESI source parameters were set as follows: Drying gas temperature (Gas Temp): 500 °C; Curtain gas: 25 psi; Collision gas: 10 psi; IonSpray voltage (IS): 4500 V; Atomization temperature: 500 °C.

*1.5. Data Analysis*

Using MultiQuant 3.0.3 analysis software, a standard curve was constructed via the internal standard method based on the response of the standard sample and its corresponding concentration, enabling the calculation of the sample concentration.

**Declaration**: The detailed procedures and methods of this study have been described in the supplementary materials of another article by our research team[1].

1.	Xu B, Chen X, Chai J, Wei Y. Dopamine Is a Key Regulatory Molecule for Escherichia coli and May Serve as a Xenosiderophore. Microorganisms. 2026;14(2); doi: 10.3390/microorganisms14020327.
