# Supplementary material for: The SaeRS two-component system likely mediates the major dopamine-dependent regulation of virulence in Staphylococcus aureus ATCC 25,923
Source: BMC Microbiol. 2026 Aug 18;26:1005. doi: 10.1186/s12866-026-05427-4 (PMC13760889; doi:10.1186/s12866-026-05427-4)
Supplement: Supplementary file 1 [file 12866_2026_5427_MOESM1_ESM.zip › Supplementary material/Supplementary Figures and Tables.docx]

Table S1

Materials required for gene knockout

| Name | Sequence 5'-3' | Source |
| --- | --- | --- |
| saeS  knock out up F | cactcatcgcagtgcagcggGGATTTACGCTGAAAGGTTTAG | Sangon Biotech |
| saeS  knock out up R | CACCATTATCGGCTCCTTTC | Sangon Biotech |
| saeS  knock out down F | GAAAGGAGCCGATAATGGTG TCCGATTTATTTATAAAATAAACTGCAAAG | Sangon Biotech |
| saeS  knock out down R | cttgcatgcctgcaggtcgaGATGAAAATTAGAGTCGTCATTCC | Sangon Biotech |
| saeS check F | GTCATGAAGTCCCTATGCGT | Sangon Biotech |
| saeS check R | GGATATTTTTCAACGATTGCAGC | Sangon Biotech |
| EcoR I | - | New England Biolabs |
| SalI | - | New England Biolabs |
| T4 DNA ligase | - | New England Biolabs |
| *E.coli* DC10B | - | Wengene Biosciences |
| S. aureus RN4220 | - | Wengene Biosciences |
| pBT2 | - | Wengene Biosciences |

Fig. S1 Functional classification analysis of differentially expressed virulence and drug resistance genes. A, VFDB annotation analysis; B, CARD annotation analysis.

Fig. S2 Verification of *saeS* gene knockout in *S. aureus* ATCC25923 by agarose gel electrophoresis. The full length of the *saeS* gene is 1056 bp. Lane A: WT strain, with a band size of approximately 1.5 kb; Lane B: *saeS* gene complete knockout strain, with a band size of approximately 0.5 kb. The leftmost lane is the DNA marker.

Fig. S3 Relative expression abundance of genes under different dopamine concentrations. RNA-seq data included samples from the 400 μM dopamine treatment group and WT control group without dopamine addition; while data in WT+100 were obtained by qPCR, with the 100 μM dopamine addition group as the treatment group and the WT group without dopamine addition as the control group.

Fig. S4 Relative gene expression levels of the WT and *saeS* knockout strains under treatment with 100 μM dopamine. The triangle symbol △ represents the *saeS*-knockout strain of *S. aureus* ATCC 25923. In the qPCR assay, WT+100, △, and △+100 indicate three treatment groups: WT supplemented with 100 μM dopamine, knockout strain treated with sterile water, and knockout strain supplemented with 100 μM dopamine, respectively. The WT strain cultured without dopamine but with an equal volume of sterile water was used as the unified control for all groups.

Table S2

Detection of the expression levels of *agrABC* and *hld*.

| Genes | WT | WT+50 | WT+100 | △ | △+50 | △+100 |
| --- | --- | --- | --- | --- | --- | --- |
| *agrA* | 1.00 | 1.22 | 1.30 | 0.78 | 1.32 | 1.42 |
| *agrB* | 1.00 | 1.36 | 1.38 | 1.04 | 1.31 | 1.50 |
| *agrC* | 1.00 | 2.19 | 2.12 | 0.92 | 1.84 | 1.96 |
| *hld* | 1.00 | 1.45 | 1.69 | 1.11 | 1.37 | 1.41 |

Fig. S5 Effects of different treatments on the growth of *S. aureus* ATCC 25923. (A) Co-incubation of bacteria with iron and dopamine, both at a concentration of 100 μM; (B) Co-incubation of bacteria with DFO and dopamine, both at 100 μM; (C) Growth profiles of the *saeS*-knockout strain with dopamine treatment. WT and Control groups were treated with an equal volume of sterile water as blank controls.

Fig. S6 Dopamine affects the generation time and CFU of *S. aureus* ATCC 25923. (A) Generation time. Labels a, b, c, and d denote the intervals 1–1.5 h, 1.5–2 h, 2–2.5 h, and 2.5–3 h, respectively; generation time was calculated from OD_600_ readings at 0.5 h intervals. (B) CFU. Control, sterile water (equal volume); DA, 100 μM dopamine.

Fig. S7 Hemolytic activity of the wild-type and Δ*saeS* strains under dopamine treatment. PC, positive control; NC, negative control.

Fig. S8 Plate assays of hemolysis, protease activity, and swarming motility. (A) Hemolytic activity; (B) Protease activity; (C) Swarming motility. Bacterial cells were inoculated onto the center of plates and cultured statically. The phenotypic results were quantified by calculating the ratio of the outer diffusion or hydrolysis zone to the inner colony diameter.

Fig. S9 Effects of dopamine on swarming motility of *S. aureus*. The Control group was treated with an equal volume of sterile water.

Fig. S10 Effects of dopamine on virulence- and colonization-related phenotypes in the WT and Δ*saeS* strains. The symbol Δ represents the *saeS*-knockout strain constructed from the WT strain. Sterile water was used as the control. (A) Hemolysis rate (5-fold dilution); (B) Enterotoxin production (5-fold dilution); (C) Protease activity; (D) Biofilm formation; (E) Chemotaxis; (F) Swarming motility. Values labeled with the same lowercase letters indicate no significant difference, while different letters indicate a significant difference at *p*< 0.05.

Fig. S11 Effects of CP and Phent on the growth of *S. aureus*. (A) WT; (B) Δ*saeS*; (C) WT; (D) Δ*saeS*. The control group was supplemented with an equal volume of sterile water, and the final concentration of DA, CP and Phent in other treatment groups was 50 μM.

Fig. 12 Swarming plate assays of *S. aureus* treated with 50 and 100 μM dopamine. Observations were performed at 18 h after inoculation. The WT and Δ*saeS* strains treated with an equal volume of sterile water served as the control groups. Bacterial suspensions were serially diluted from undiluted stock to 10⁵, and spotted on each plate from left to right.

Table S3

Functional description of selected ROS‑related genes.

| Gene Name | Functional Description |
| --- | --- |
| *ahpC* | Removes intracellular peroxides to resist oxidative stress |
| *ahpF* | Cooperates with AhpC to clear reactive oxygen species |
| *recA* | Triggers SOS response to repair damaged DNA |
| *lexA* | Inhibits SOS gene transcription without DNA damage |
| *uvrA* | Identifies oxidative DNA damage sites |
| *uvrB* | Takes part in nucleotide excision repair |
| *dinB* | Enables DNA replication across damaged sites |

Fig. S13 ROS levels in *S. aureus* after different treatments. (a) Control group treated with an equal volume of sterile water; (b, c) groups treated with 100 μM and 400 μM dopamine, respectively; (d, e) groups treated with 100 μM and 400 μM H₂O₂, respectively. Intracellular ROS levels in *S. aureus* were measured using the Bacterial ROS Assay Kit (BestBio, Shanghai, China; catalog number BB‑461114). Consistent with the transcriptome sample preparation, 400 μM dopamine was added to the culture, and after incubation at 37 °C with shaking at 180 rpm for 30 min, the bacterial cells were harvested by centrifugation. The cells were washed three times with PBS and then incubated with BBoxiPro® O14 fluorescent staining working solution for 30 min at 37 °C in the dark. After incubation, the cells were washed again with PBS, resuspended, and transferred to a black 96‑well plate. Fluorescence intensity was measured using a fluorescence microplate reader at an excitation wavelength of 658 nm and an emission wavelength of 676 nm. Intracellular ROS levels were expressed as relative fluorescence intensity compared with the untreated control. Each assay was performed in triplicate.

Fig. S14 Transcript levels of selected genes in *S. aureus* following different treatments. Control: sterile water (equal volume); DA, dopamine at 100 or 400 μM.
