# Supplementary material for: The SaeRS two-component system likely mediates the major dopamine-dependent regulation of virulence in Staphylococcus aureus ATCC 25,923
Source: BMC Microbiol. 2026 Aug 18;26:1005. doi: 10.1186/s12866-026-05427-4 (PMC13760889; doi:10.1186/s12866-026-05427-4)
Supplement: Supplementary file 1 [file 12866_2026_5427_MOESM1_ESM.zip › Supplementary material/SupplementaryOmics Data.docx]

Supplementary Omics Data

| Raw dataset type | Repository | Bioproject ID | Accession ID | Direct Link |
| --- | --- | --- | --- | --- |
| RNA-seq | NGDC- GSA | PRJCA063171 | CRA042114 | https://ngdc.cncb.ac.cn/gsa/browse/CRA042114 |
| DIA proteomics | ProteomeXchange Consortium |  | PXD078195 | https://proteomecentral.proteomexchange.org |
| Untargeted metabolomics | NGDC- OMIX | PRJCA063171 | OMIX016583 | https://ngdc.cncb.ac.cn/omix/release/OMIX016583 |
